# Lipoproteins comprise at least 10 different classes in rats, each of which contains a unique set of proteins as the primary component

**DOI:** 10.1371/journal.pone.0192955

**Published:** 2018-02-20

**Authors:** Tomokazu Konishi, Yoko Takahashi

**Affiliations:** 1 Graduate School of Bioresource Sciences, Akita Prefectural University, Akita, Japan; 2 Division of Food Function Research, Food Research Institute, National Agriculture and Food Research Organization (NARO), Tsukuba, Ibaraki, Japan; Universita degli Studi di Milano, ITALY

## Abstract

Although lipoproteins are conventionally separated into a few classes using density gradient centrifugation, there may be a much higher number of physical classes that differ in origin or phase. Comprehensive knowledge of the classes of lipoproteins is rather limited, which hinders both the study of their functions and the identification of the primary causes of related diseases. This study aims to determine the number of classes of lipoproteins that can be practically distinguishable and identify the differences between them. We separated rat serum samples by gel filtration. The elution was continuously monitored for triglyceride (TG), cholesterol, and protein, and fractionated for further SDS–PAGE and immunological detection of apoprotein A-I (ApoA1) and apoprotein B (ApoB). The elution patterns were analyzed using a parsimonious method, i.e., the estimation of the least number of classes. Ten classes were recognized that contained different amounts of TG and cholesterol, as well as a unique protein content. Each of the classes contained much more protein than that observed previously, especially in low-density lipoproteins (LDL) classes. In particular, two major antiproteases formed complexes with specific classes of LDL; because these classes exclusively carry cholesterol and antiproteases, they may lead to the progression of atheroma by supplying materials that enlarge fatty streaks and protecting thrombi from enzymatic digestion. The separated classes may have specific biological functions. The attribution of protein species to certain classes will help understand the functions. A distinction among lipoprotein classes may provide important information in the field of vascular pathology.

## Introduction

Animals retrieve lipids from foods, or synthesize them in the liver and supply them to the whole body through the bloodstream [1–4]. As lipids are insoluble in aqueous plasma, they are carried in complex particles known as lipoproteins, which include phospholipids (PL) and proteins that may stabilize the emulsion as surfactants [5].

Lipoproteins have been classified according to their size, content, and/or biological role. The classes are traditionally isolated using density gradient centrifugation in biochemical studies [6–10] and clinical measurements [11]. This is a rather unique process that repeats a series of isopycnic centrifugations. The densities of samples are adjusted using dense mediums, such as high concentrations of salts or D_2_O and sucrose, and the gradient is autonomously formed under high gravity. Subsequently, very-low-density lipoproteins (VLDL), LDL, and high-density lipoproteins (HDL) are collected step-by-step by repeating the gradient-formation process using more concentrated media.

Although this method is also used to measure the classes, the process is very lengthy. Hence, many alternative methods have been developed to quickly reproduce the results of density gradient centrifugation (reviewed in Okazaki and Yamashita, 2016) [12]. Gel filtration, which is one of these alternatives separates particles according to size: while passing through porous gel beads packed into a column, smaller particles will enter the pores of the beads more easily, thus flowing over longer distances and exhibiting greater elution times.

However, the analysis of gel filtration also lacks maturity, as it does not include a critical biochemical background. The elution patterns are interpreted using curve-fitting methods; by estimating several lipoprotein classes, patterns are summarized using a set of parameters, as follows. This method is based on several characteristics of the physical behavior of particles in a column; first, the elution time of the pure material is normally distributed. As a particle faces many choices regarding whether to enter the gel beads or not, the flow distance will be the sum result of such random choices; according to the central limit theorem, this will be normally distributed. In fact, the peaks corresponding to pure materials, such as glycerol, are well approximated by a normal distribution. In particular, particles that are larger than the pores elute constantly and are normally collected in a fraction called void. The two parameters of normal distribution, location and scale, of a particle with a certain size are found as constants of each gel column, indicating the time and range of a peak, respectively. Second, the elution time is proportional to the logarithm of the particle size. This could be due to the physical properties of the gel beads; i.e., the size distribution of the pores. In addition to the properties of pure materials, because a class of lipoprotein is composed of particles with different sizes, the population of proteins also affects actual patterns. Empirically, the elution time of a class is normally distributed and exhibits a much wider scale than do pure materials. This property shows that the diameters (hence, the volume) of a lipoprotein class vary greatly and are lognormally distributed. This distribution pattern is reasonable for physical properties found in typical emulsion [13]. In each class, the patterns of TG and cholesterol share scale and location; this shows that, despite the size variation, the ratio of TG to cholesterol is constant in a class. As serum is a mixture of several classes of lipoproteins, the recorded elution patterns are composites of peaks of such classes; thus, the size ranges among some classes are physically overlapped. Based on scale and location, the patterns of TG and cholesterol are fitted using a third parameter (quantity of the content).

The curve-fitting method critically depends on how many classes are expected in the serum; for example, a method that estimates 20 classes is widely used [12]. This method uses fixed location and scale values that were determined based on patterns of samples, some of which are uniquely found in human diseases. Although the large number of classes can sufficiently fit various patterns, there is a concern regarding overfitting the model: because no biochemical evidence has been supplied, whether such classes exist physically and the necessity of the parameters are aspects that remain uncertain. In particular, the application of the parameters to other species, such as rodents, which exhibit patterns that are very different from those of humans, may not be reasonable.

Recently, a proteomics approach using mass spectrometry (MS) was applied to human plasma and disclosing many new components [14–17]. Because many of those research efforts aimed to identify markers of diseases, they did not always study the attribution of the proteins; however, several studies targeted certain classes of lipoproteins that were isolated by density gradient centrifugation [9, 18], gel filtration [10], a mixture of both [19, 20], or immunocapture [21]. Studies often separate lipoproteins into three classes: VLDL, LDL, and HDL. The attributions of the proteins did not necessarily match among the studies. This conflict might have been caused partly because of the limited quantitative ability of MS [22]; compensation using internal standards is essential [23], but it is difficult to prepare these standards in advance. This limitation hampers the determination of whether a protein is a major component or a contaminant within a lipoprotein class. Another possible explanation is that lipoproteins isolated using different procedures might contain different components, as will be discussed later. Nevertheless, to identify the functions of the proteins detected, as well as the corresponding lipoproteins, their attributions are critically important, and this requires a reliable method for the segregation of lipoprotein classes.

The classification of lipoproteins is also important for identifying the primary cause of certain diseases. For example, as cholesterol is one of the major components of atheroma, LDL may play an important role in the progress of atherosclerosis [24, 25]. However, the 2013 American College of Cardiology / American Heart Association guideline could not specify the LDL and non-HDL cholesterol goals that are necessary to prevent atherosclerotic cardiovascular disease [26], in spite of several huge epidemiology studies have been performed. Still, the quantification of cholesterol classes remains important in clinical practice [27, 28]; at present, the level of LDL cholesterol is used for diagnosis, and the determination of specific cholesterol or apolipoprotein goals is encouraged as a future research need by the ACC/AHA guideline [26]. Undoubtedly, the identification of classes of lipoproteins that cause specific diseases is important for their treatment and prevention.

We collected and separated sera from rats using gel filtration, and studied the fractions biochemically. To monitor protein patterns in the elutes, UV absorption was recorded in addition to cholesterol and TG; the three patterns were fitted with least number of classes. The eluted lipoprotein patterns were interpreted by estimating 10 classes of lipoproteins, each of which was well fitted by a normal distribution. Moreover, apoprotein A-I (ApoA1) and and apoprotein B (ApoB), which are the main components of HDL and LDL, respectively, were checked immunochemically, and other proteins were verified via SDS–PAGE. In addition to correcting the misidentification of lipoprotein classes reported in rat studies [29], these results showed that the protein content of all lipoproteins was much higher than that observed previously, which raises questions regarding classification based on differences in density.

## Materials and methods

### Animals

Six male Sprague Dawley rats (4 weeks, 143–147 g) were housed individually in controlled temperature (20–22°C), humidity (55–65%), and 12-h-light/dark cycle, and were acclimatized to a commercial food (Type NMF; Oriental Yeast, Tokyo, Japan) for 5 days. Animals were fed an experimental diet based on AIN-93G [30] that contained casein (Wako Pure Chemical Industries, Osaka, Japan) or dehydrated soybean curd (kori tofu; Misuzu Co., Ltd., Nagano, Japan) as protein source, and soy oil (Wako Pure Chemical Industries) or fish oil (NOF Corporation, Tokyo, Japan) as fat source. Animals had free access to the diets and tap water for 21 days. The animal study was approved by the Review Board of Animal Ethics of the Food Research Institute, NARO (approval number: H19-052) and was performed in accordance with the institutional guidelines for the care and use of laboratory animals.

### Serum and fractionation

After 3 h of food deprivation, animals were euthanized by collecting excessive blood from the abdominal aorta under isoflurane anesthesia. Serum samples were obtained by centrifugation at 1500 g for 10 min after clotting of blood for 2 h at room temperature. Sera contained on average 0.6 g/L of both cholesterol and TG, 1.4 g/L of PL, and 80 g/L of protein (S1 Table). Serum PL, TG and cholesterol were analyzed using commercial kits (Phospholipid C-Test, Triglyceride E-test, and Cholesterol E-test Wako; Wako Pure Chemical Industries). Total protein was measured by Lowry method [31], using bovine serum albumin as the standard (Fraction V, Roche, Basel, Switzerland).

Serum samples of 10 μL were separated by high-pressure liquid chromatography (HPLC) with a gel filtration column [12, 32], giving 45 fractions (LipoFraction, Skylight-Biotech, Akita, Japan). The system separates samples under moderate ionic conditions in a short period; the void peak, which is composed of particles too large to enter the gel pores, was eluted from the system after 16.5 min, while that of glycerol was eluted at 34 min; lipids and proteins were detected between these time points. The column effluent was split equally into three aliquots: two were tested for TG and cholesterol, and one was used for UV analysis to detect proteins and then fractionated. The volume of each fraction obtained was 550 μl.

### Detection and quantification of signals

Proteins were visualized on SDS–PAGE. A 12 μl sample of each fraction was mixed with Laemmli sample buffer (Bio-Rad Laboratories, Inc., Hercules, CA) supplemented with β-mercaptoethanol, non-heated, and separated using a Mini-Protean TGX Precast Gel (4–15%), with Precision Plus unstained standards (Bio-Rad Laboratories). Proteins were detected using SYPRO Ruby Protein Gel Stain (Ronza Rockland, Inc., Rockland, ME). Fluorescence signals from the antibodies and proteins were detected, recorded and measured using a Typhoon FLA 9000 (GE Healthcare), and was quantified by scanning the images using ImageJ [33]. The major bands in fractions 19 and 23 were excised from the gel and identified by matrix assisted laser desorption/Ionization-time of flight mass spectrometer (MALDI-TOF MS) (Genomine Inc., Kyungbuk, Republic of Korea) and analyzed through the database of Matrix Science Inc. (Boston, MA).

ApoB and ApoA1 proteins were detected in each of the fractions by slot blotting 15 and 6.25 μl samples, respectively, of the fractions onto PVDF membrane (Hybond-LPF, GE Healthcare, Pittsburgh, PA) using a Bio-Dot^®^ SF Apparatus (Bio-Rad Laboratories). The blotted proteins were reacted with goat anti-ApoB antibody (1:1000; sc-11795, Santa Cruz, Dallas, TX) and anti-goat IgG Cy3 conjugate (1:5000; AP180C, Merck Millipore, Darmstadt, Germany), or rabbit anti-ApoA-I antibody (1:1000; sc-30089, Santa Cruz) and ECL Plex goat-anti-rabbit IgG-Cy5 (1:5000; PA45012, GE Healthcare).

### Curve fitting

The components of the lipoprotein, TG, cholesterol, and protein, in the HPLC effluent were recorded continuously over time. Curve fitting was performed on the recordings using a parsimony model (see legend of S1 Fig). Ten classes of normal distributions were used to fit lipoproteins, and additional two classes were used for albumin and free glycerol. Lipoprotein particles of each class could vary in size but contained uniform proportions of components. The size variation was log-normally distributed, so each class could be represented in the HPLC recordings by a normal distribution that had specific parameters for location and scale. The location represents the peak time, and the scale represents how the sizes of particles varied within a class; these parameters are denoted in the text as time and range, respectively. Using these parameters, the probability density functions of the normal distributions were multiplied by the magnitude parameters to fit each of the components’ recordings using the *optimize* function of R software [34].

The coincidence of peak time and range between TG, cholesterol, and UV traces was confirmed as follows. Full sets of the time and range parameters for a sample were first identified using the components indicated in S2 Table (profile). Then, the time and range parameters for each class were re-estimated one by one using the other components’ recordings. Differences found between the parameters for TG, cholesterol, and UV were evaluated for each sample in the form of variance; the square root of the mean variance between samples, representing the average SD of the estimations, is presented in S2 Table. Estimation of time and range parameters for the UV traces for LDL2 and mature form of HDL (mHDL) [35] was not possible because of interference by the neighboring strong signals from anti-proteases and albumin.

### Proportion of the components

The composition of each class of particles was estimated as the mean of the w/w percentage of the six samples. Some missing values were estimated based on observations of samples separated by centrifugation, as described by Oschry & Eisenberg [8]. PL was measured as the total value and was divided into classes according to the spherical surface per volume of the particles (see legend of S8 Table). The ratio of esterified (CE) and free (UC) cholesterol for each class was estimated according to Oschry & Eisenberg [8].

The densities of the particles were estimated as the weighted average of their components; the volume of a mixture will be the sum total of the components. The densities used for CE, UC, TG, PL, and protein were 0.95, 1.05, 0.913, 1.00, and 1.47 g/ml [36], respectively. Assuming that the particles were spherical, the mean molecular weight of a class was further estimated using its estimated density and measured size. Its concentration was derived from its molecular weight and the sum of the components.

The concentrations of the 15- and 10-nm LDL-like particles, that were not attached to the antiproteases, LDL3 and LDL4, were estimated as if they contained the same percentage of proteins as LDL1 and LDL2, respectively (S9 Table). The numbers of molecules per particle were estimated from their composition.

## Results

**Fig 1:**
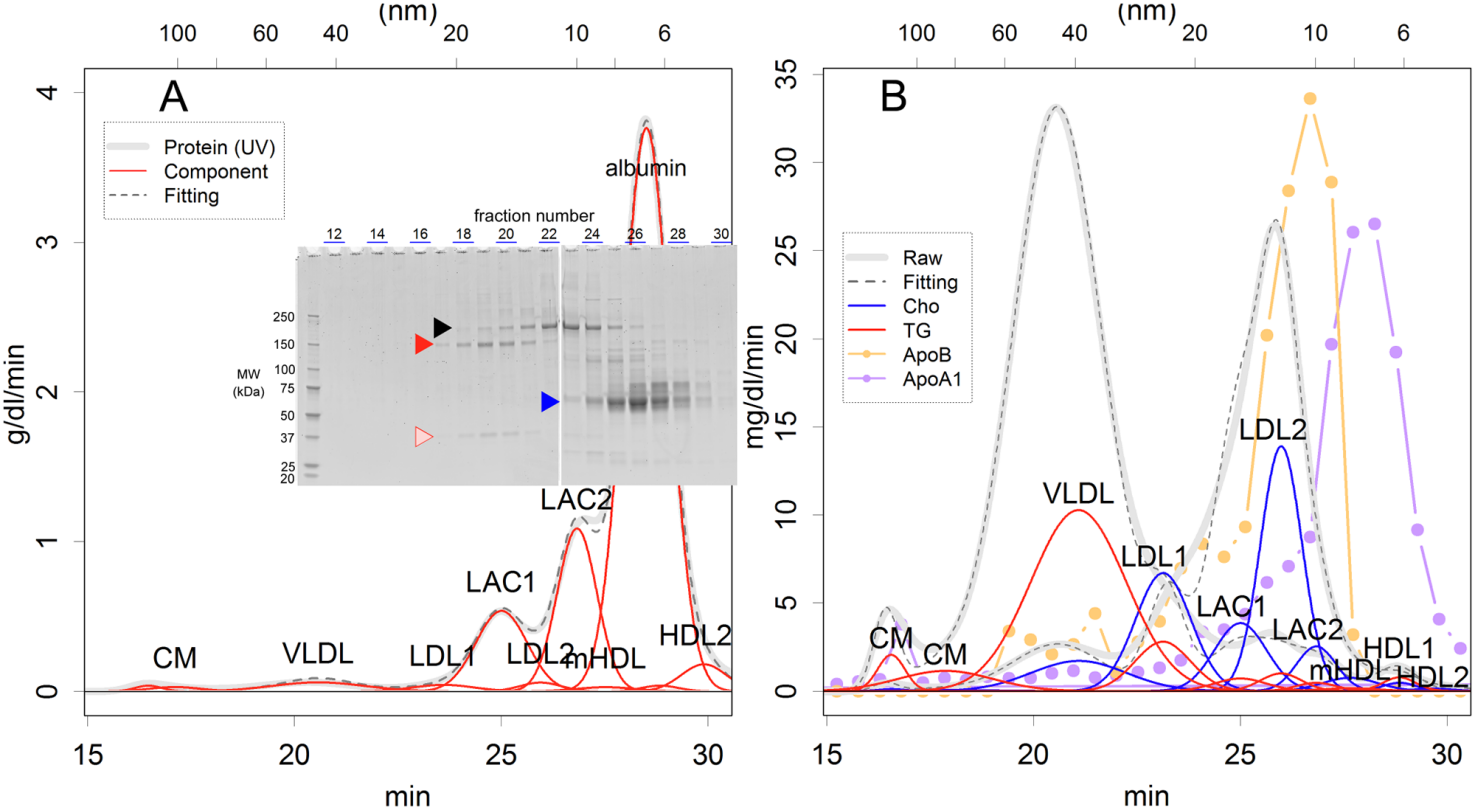
Elution patterns of the gel filtration chromatography. **(A)** Pattern of UV absorption for monitoring proteins. The positions of fraction numbers on SDS–PAGE analysis correspond to the time on the UV absorption curve. The logarithm of the particle size and the elution time were proportional (S2 Fig). The arrowheads show bands for A1i3 (black), A1m (red and pink), and albumin (blue). (**B**) Patterns of TG (red) and cholesterol (blue). Evidence from slot blots for ApoA1 and ApoB is also presented (S9 Fig, relative values). Raw: monitoring record; Fitting: sum of the fit curves. Abbreviations for lipoprotein classes are given in the legend of Table 1.

The elution patterns of the lipoprotein components cholesterol, TG, and protein could be well approximated using 12 sets of normal distributions: 10 for lipoproteins, albumin and free glycerol (Fig 1; attribution of the classes will be discussed later, Table 1, evidence for the number of parameters is shown in S1 Fig, relationship of size and elution time in S2 Fig). Curve fitting was performed by identifying the time and range parameters for each distribution, and then a parameter of magnitude was determined for each component. The time and range parameters identified for cholesterol, TG, and protein differed only slightly (S2 Table), showing that they reflected signals from identical particles that were separated by the column. The isolated peak of free glycerol had a range of 0.29 min, and this may indicate the noise level of the system for measuring the size of single molecule. The fitting method was developed parsimoniously (S1 Fig), and could fit samples taken from variously fed animals well (S3 and S4 Figs), although the contents of the sera differed much (S1 Table). The time parameters were constant among samples, showing that the amount, but not the size, of lipoproteins differed among samples (Table 1, fluctuations). Albumin eluted slightly earlier than did HDL1 (Table 1), thus overlapping with other polypeptides (S5–S7 Figs).

**Table 1:** Parameters used to fit the classes of lipoproteins.

	CM(void)	CM	VLDL	LDL1	LAC1	LDL2	LAC2	mHDL	HDL1	HDL2	alb	glyc
Time	16.5	17.6	20.8	23.3	25.0	26.0	26.8	27.6	28.8	29.8	28.5	34.0
Range	0.38	0.48	1.13	0.69	0.63	0.50	0.49	0.65	0.42	0.54	0.56	0.29
Diameter	118	97	42	23	15	12	10	8.4	6.2	4.9	6.6	1.8
Fluctuation	0.05	0.17	0.24	0.17	0.02	0.03	0.02	0.18	0.08	0.09	0.02	0.00

The time and range parameters (min) were identified from the location and scale, respectively, of the normal distributions. The diameter (nm) of the particles was estimated from S2 Fig. The fluctuation (min) indicates differences between samples as the standard deviation (SD) of the time parameter. CM, chylomicron; CM(void), chylomicron in the void; VLDL, very-low-density lipoprotein; LDL, low-density lipoprotein; HDL, high-density lipoprotein; alb, albumin; glyc, glycerol; LAC1 is a complex of hypothetical LDL3 (S9 Table) and A1m, while LAC2 is a complex of LDL4 and A1i3. All sets of parameters used are shown as S6 Table.

Two major protein inhibitors, alpha-1-macroglobulin (A1m) and alpha-1-inhibitor 3 (A1i3), were detected on SDS–PAGE of the fractions (Fig 1A and S6 Fig) and identified using MALDI-TOF MS (S8 Fig). They showed clear peaks on the UV traces, which coincided with those of cholesterol and TG (Fig 1B), suggesting that they would be components of classes of lipoproteins. It is noteworthy that these complexes may worsen atheroma, as will be discussed later.

These normal distributions are likely to represent 10 classes of lipoproteins separated by the gel column, plus albumin and free glycerol (Fig 1). Their distribution may reflect the nature of the micelles: the diameters of dispersed micelle particles were log-normally distributed [13], and such particles would be eluted from the column in a normal distribution.

A large fraction of serum proteins was a component of certain lipoproteins. The record of the UV pattern may reflect the amount of proteins appropriately; in fact, the patterns coincided well with the Lowry method and SYPRO Ruby staining of SDS–PAGE (S10 Fig) of the fractionated sample. The distribution parameters corresponded well with those of TG and cholesterol (S2 Table). The location and scale parameters could not be estimated for some of the lipoproteins, because of disturbance of major proteins (S3 and S4 Tables); however, the parameters found from lipids fit the UV records as well (Fig 1A and S3 and S4 Figs). In fact, the failure of fitting was only 3% of the total record in the range between 15 and 30 min of elution (S5 Table). These tight coincidences of location and scale parameters (S2 Table) show that most of the proteins eluted were exactly concordant with lipoproteins, with the exception of albumin. This is possible if and only most of the proteins are components of lipoproteins in the range; the amount of proteins that are free from lipoproteins would be limited in serum.

The primary components of lipoproteins were proteins. The protein bands detected in SDS–PAGE seemed to behave similarly to certain classes of lipoproteins (Fig 1A, S7 Fig and S7 Table). In addition, the amount of proteins in the serum (80 g/L, S1 Table) was much greater than that of TG or cholesterol (0.6 g/L); therefore, the primary component of lipoproteins seems to be proteins, not lipids. Thus, the composition of each class of lipoproteins was estimated, showing that they contained much more protein (Table 2) than did the previous estimates for LDLs, of 2%–24% [1–3]. It should be noted that many of the lipoprotein classes would have similar densities.

**Table 2:** Estimated composition of the particles.

		CM	VLDL	LDL1	LAC1	LDL2	LAC2	mHDL
Composition (%)	TG	4.7	20	4.1	0.16	0.92	0.057	0.58
	CE	0.22	1.5	12	2.1	18	0.44	3.7
	UC	0.34	2.5	3.4	0.58	5.1	0.12	0.52
	PL	1.2	13	15	3	32	1	9.2
	Protein	93	62	65	94	43	98	86
	density	1.43	1.28	1.3	1.44	1.2	1.46	1.4
	conc. (nM)	0.78	67	210	4,400	2,200	26,000	2,600
Molecules/particle	TG	41,000	6,800	230	3.1	7.2	0.29	1.7
	CE	5,200	1,200	1,600	89	330	5.2	25
	UC	4,900	1,100	260	15	54	0.85	2
	PL	12,000	5,300	1,000	67	300	6.3	32
	Protein	1,400^a^	37^a^	6.5^a^	1.4^b^	1.2^b^	1.1^b^	7.8^c^

The average of six samples is shown. The proportions of PL, cholesterol esterified (CE), and unesterified cholesterol (UC) were derived from the results of centrifugation [8]. Density was derived assuming that mixing of components would not affect the total volume. Conc: concentrations derived from the estimated densities. CM and CM(void) were summed. Because the proteins of HDL1 and HDL2 could be cross-contaminated with albumin, they were omitted from the table. Protein represents the estimated number of molecules contained in a particle.

^*a*^ Estimated protein of 500 kD.

^*b*^ LAC1 was estimated as the number of complexes of a single ApoB100 and a tetramer of A1m, LDL2 was estimated as a monomer of ApoB48, and LAC2 was estimated as a heterodimer of B48 and A1i3.

^*c*^ Estimated as a monomer of ApoA1.

Contents of PL were estimated according to S8 Table.

Next, we identified the classes of lipoproteins based on the three known pathways of lipoprotein production [1–4]. In this way, it is possible to comprehend lipoproteins not by their size, but through their functions. Of these pathways, two supply TG and cholesterol to the body; one of which starts with chylomicron (CM) secreted from the intestine to lymph, while the other starts with VLDL that are secreted from the liver. Both pathways lead to production of smaller LDLs by the loss of TG, which is degraded by lipoprotein lipase (LPL) located on the surface of blood vessels. The third pathway removes excess cholesterol from tissues: HDL are secreted from the liver as small precursors and become larger by incorporating cholesterol. Each type of lipoprotein is composed of specific apoproteins: CM, VLDL, and their derivatives are based on monomeric ApoB100 or its truncated version ApoB48 [37, 38], while HDL is wrapped in a shell of dimers or oligomers of ApoA1 [35]. These supply and recovery pathways are clearly separated in rats: although cholesterol can be transferred between HDL and LDL in many species, rats lack the enzyme that facilitates this exchange [8, 39]. To verify the attributions, the main components of LDL and HDL, ApoB and ApoA1, were immunochemically detected from fractionated samples using a slot blot analysis (Fig 1B and S9 Fig). Disturbance by nonspecific binding of antibodies was limited, as none of the antibodies responded to the major protein components: the two protease inhibitors and albumin (Fig 1).

CM eluted from our column as twin peaks: the earlier peak appeared at the void of the column (Table 1, Fig 1); thus, the validity of data is rather limited. However, in the later peak, the time and range parameters for UV, cholesterol, and TG coincided well (Fig 1, S2 Table), suggesting that the signals reflect substances that are included in a lipoprotein class. The estimation of the amount of material showed that the class contained a large quantity of proteins, which was distinctively different from what we predicted (Table 2) [1–3]; however, the missing proteins have not been identified.

**Fig 2:**
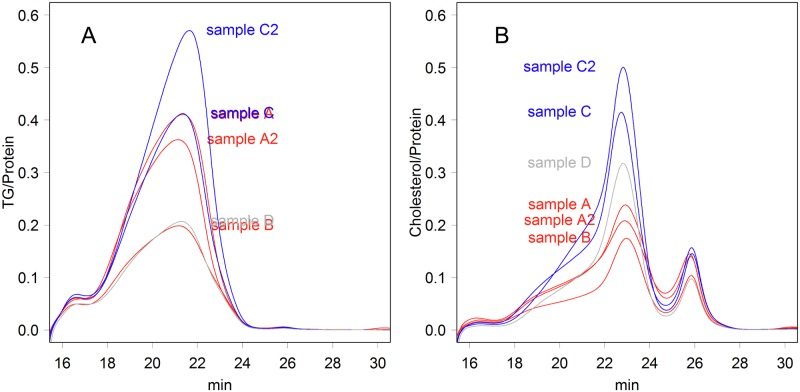
Ratios of lipid and protein in the samples. (**A**) TG to protein (w/w). **(B)** Cholesterol to protein (w/w).

The size of VLDL was variable, showing a wider range (Table 1). The time parameter also varied between samples (Table 1, fluctuation). These particles may have a flexible cargo capacity for lipids, especially for TG, and this latitude may have increased these parameters. Such latitude was also observed in the amount of proteins and lipids: the ratio of protein to TG or cholesterol varied greatly between samples, suggesting that different body conditions that might have been provoked by the feedings (Fig 2).

LDL should contain high levels of cholesterol and less TG. Two classes of particles met these criteria (Table 2, LDL1 and LDL2). It was believed that HDL is the main carrier of cholesterol in rat serum; the two main peaks in the cholesterol pattern had been believed to be LDL and HDL [29]. However, the distribution of ApoB as well as ApoA1 showed that they were VLDL and composite of LDLs (Fig 1B).

Two anti-proteases, A1m (Fig 1A, red arrowhead) and A1i3 (black arrowhead), formed complexes with ApoB and cholesterol that we denoted LDL–anti-protease complex (LAC). These major proteins showed clear peaks on the UV traces (Fig 1A and S10 Fig). The time and range parameters for these proteins were practically identical to those found for TG and cholesterol in specific LDL-like particles (LAC1 and LAC2 in Fig 1, and LDL3 and LDL4 in S9 Table; LDL3 and LDL4 are hypothetical particles that have the same size as LAC1 and LAC2, respectively). These similarities in the parameters could not have occurred by chance; the range was much larger than the noise level (that of glycerol: i.e., 0.29), showing that the diameters of the proteins were indeed log-normally distributed, which is difficult to envisage as the characteristic of a single protein. Additionally, ApoB was detected in the corresponding fractions (Fig 1B). If the LDL-like particles did not contain anti-proteases, the huge particles must have been filled with cholesterol, meaning that the number of particles would become much lower (S9 Table). This conflicts with the pattern of the ApoB signal (Fig 1B); if this were the case, the signal would be much broader, with a peak at LDL2.

Three classes of lipoproteins were detected together with ApoA1, the apoprotein of HDL (Fig 1B). Among these three classes, the largest was consistent with mHDL that has absorbed cholesterol from peripheral tissue [35]. The other classes may represent putative discoidal HDL, which can accept cholesterol (Table 1, HDL1 and 2) [4].

## Discussion

In continuous records of TG, cholesterol, and UV, 10 classes of lipoproteins were found based on the elution patterns of gel filtration analyzed by curve fitting. Each of the protein bands was detected from specific fractions of gel filtration (Fig 1A), suggesting that the classes of lipoproteins are distinct not only in their size, but also in protein content. Therefore, each of the classes may have unique biological functions. The number of classes was required for curve fitting, and there could be more classes in a physical context. For example, VLDL is most likely a mixture of particles based on ApoB100 and ApoB48, and each of the core proteins may exhibit different size distributions. In fact, the range of VLDL was much larger than that of other particles, and the elution time fluctuated (Table 1); each of these estimated subclasses may have a stable elution time and narrower range. Additionally, there could be more subclasses that have different functions, containing specific sets of proteins; Gordon et al. (2010) [10] compared the fractions obtained by ultracentrifugation and gel filtration, and showed that the latter possessed much more protein species (see, for example, Fig 2 of the reference). Testing this possibility may require additional separation techniques that distinguish aspects other than size of a particle.

The elution patterns of gel filtration (Fig 1) were similar to those found in previous studies [10, 19, 29], despite the fact that the gel columns or detection systems were different. For example, one-third of the proteins were detected in the size range of lipoproteins, and the highest proportion of cholesterol/protein was observed at LDL (ratio, 0.2–0.5) (Fig 2B). The dominance of proteins was also observed in human samples (S11 Fig); in fact, the elution pattern and data range were similar to those presented in Fig 2B. However, the elution patterns differed from those of materials that were isolated by density gradient centrifugation; lipoproteins isolated in this way showed very sharp peaks in gel filtration [18–20], as will be discussed later.

Other than the similarities in elution patterns, the attribution of the peaks was totally different from previous studies; this difference critically asks for reevaluation of analyses, clinical guidelines, and the understanding of the classes and functions of lipoproteins. The difference is partly caused by the data-driven approach of analysis used in this research; we did not try to classify lipoproteins into predetermined systems, such as that comprising three classes: VLDL, LDL, and HDL; thus, the number of estimated classes would be inevitably different. However, there were also qualitative differences. For example, the largest peak of cholesterol was identified as some subclasses of LDL in this study (Fig 1A), whereas it was thought to be HDL in rat serum [29]. Although no evidence was presented for this “huge amount of HDL” scenario, it might have arisen to confirm the results with those of studies that used density gradient centrifugation. In our study, HDL classes were verified using an anti-apoA1 antibody, and the sizes were identical to those found in 2D PAGE [14] or estimated based on protein structure [35]. In previous studies, to broaden the assignment of HDL cholesterol, very large particles, such as those with a size comparable to LDL, were assigned as HDL; this was also done for human samples [10].

Lipoprotein samples obtained using different procedures, i.e., gel filtration and density gradient centrifugation, are qualitatively different. The differences are apparent in the following three aspects of the separated lipoproteins: protein content, amount of protein, and size distribution. First, many of the major proteins of LDL and HDL were common among samples separated by density gradient centrifugation [9, 18]. However, such common major components were rather limited among those that were purified by gel filtration (Fig 1A, S6 and S7 Figs); rather, proteins were detected from their specific fractions, and such specificity was also found in a previous study that used MS [10]. Second, lipoproteins separated by gel filtration contained a large amount of protein, and so would have a much higher density (Table 2) than that observed previously [1–3]. Third, lipoproteins isolated by density gradient centrifugation showed very sharp peaks in the subsequent gel filtration [18–20]. As found in previous studies [10] and in the current work (Fig 1), LDL and HDL of raw serum samples showed rather wider distributions, which were composed of several subclasses. Hence, sharp separation is not expected, even in gels with a greater resolution. Moreover, the width of peaks became sharper after repeating centrifugation [19], suggesting the convergence of lipoprotein sizes during centrifugation, as follows.

Density gradient centrifugation places lipoproteins under high gravity to form the density gradient [6–9, 19, 20]. As LDLs are droplets of lipids that are surrounded by a single molecule of apoB [37] and PL, they would be rather fragile in physical terms; if some proteins are attached to a class of LDL by a hydrophobic effect, the gravity force could be sufficiently large to pull the proteins off [40] (S14 Fig). If convergence and/or protein removal occur in reality, these rearrange lipoproteins to a new state with better stability in the extreme conditions. The common protein components found among lipoproteins [9, 18] could be explained by this scenario. Moreover, if a fraction of LACs or CM survived this rearrangement, they would contaminate the HDL fraction of the density gradient centrifugation. This would lead to an overestimation of HDL.

In contrast, it is possible that some non-HDL proteins were included in the HDL fractions isolated by gel filtration. In fact, some proteins are as large as the smaller lipoproteins; for example, the diameter of tetrameric IgG was measured as 6 nm by dynamic light scattering [41], and albumin was detected from a fraction at 7 nm (S7 Fig). The size of albumin, which was much larger than that of its nascent form, suggests binding to fatty acids [42]. These two examples would be the size range of HDL (Table 1). However, it is difficult to estimate free proteins that are much larger. For example, a globular protein with a size similar to that of VLDL may have a molecular weight of 33 MDa, although even the largest antibody, pentameric IgM, is less than 1 MDa. In addition, the time and scale parameters used to fit the patterns of TG, cholesterol, and UV were the same (S2 Table); such consistency cannot be expected if they did not correspond to identical particles.

The protein detected in the CM fraction may contain some contaminants, because these were larger than the resolution range of the column. The first peak was at the void of the column; hence, huge materials such as fractured blood cells, if present, would appear in the same fraction. Moreover, the column may work as a solid sieve for materials that cannot enter gel pores; although the sharing force is uncertain, it could break part of the fragile huge materials, thus also affecting later peaks. However, even after considering such artificial effects, CM may contain a large amount of protein, even though it had been thought to contain only 2% of protein, as it exhibited the lowest density among lipoproteins [1–3]. Actually, CM could have been missed in previous studies using centrifugation: the lightest fraction in those studies contained particles of diameter ca. 35 nm [7, 8], which is much smaller than that of CM found in lymph [43] or in this study (Table 1). Therefore, CM are likely to contain many totally unknown proteins that carry the huge lipidic particle through thin capillary tubes.

We propose that the composition of LACs is as follows. The anti-proteases belong to the A2m gene family, which is known to be expressed upon infection; rabbit A1m is a tetramer [44], and rat A1i3 is a monomer protein [45]. It is possible to predict from the particle size that LAC2 may be a heterodimer of A1i3 and ApoB48. Assuming that it has a spherical shape, the density of such a heterodimer would be 1.3, leaving space for a few molecules of cholesterol; this is very consistent with the estimation derived from the monitored profiles (Table 2). The larger size of LAC1 allows several possible combinations; however, the well-studied A2m forms a tetramer of 14–18 nm diameter, and the purified protein has a large cavity [46]. It also forms a complex with cholesterol acyltransferase [47], a component of LDL. As a housekeeping version of A2m, if a tetramer of A1m formed a complex with ApoB100 and several other apoproteins, it would still have plenty of cargo space for cholesterol, which is consistent with the particle size listed in Table 2. Because the antiproteases were not found as free form in serum (Fig 1A, S6 and S7 Figs), LACs may be formed where the antiproteases are synthesized. Additionally, as LAC1 and LAC2 differ in size, range, and anti-protease content, they are likely to be formed in different cells of the liver. Liver cells could synthesize components of LACs de novo; however, ApoB100 or B48, as well as cholesterol, could also be salvaged from LDL recovered by liver.

LACs may have special functions. Those are complexes of protease inhibitor and cholesterol (Fig 1 and Table 1). The protease inhibitors would protect clots by disturbing fibrinolysis, thus facilitating coagulation. Moreover, the incorporated cholesterol would supply building blocks for wound repair. Conversely, they may be complicit in progressing atheroma by supplying materials that enlarge fatty streaks and protecting thrombi from enzymatic digestion; those would be the LDL particles that supply lipids to plaques [24, 25]. Corresponding classes would exist in humans as well; for example, A2m was detected as a likely counterpart of smaller LDL [10], although it was also found in the size range of HDL as a complex with LCAT [47]. Nevertheless, the antiproteases observed in proteomics studies [9, 10, 13–21] may form specialized classes of lipoproteins; if so, those would be important clinical drug targets that should be measured. As the ratios varied greatly among samples (Fig 2), the amount of both cholesterol and protein should be measured in studies of the classes of lipoproteins.

Although the separation of gel filtration is fine (Fig 1), in a practical sense, the method could not handle many clinical samples. Therefore, identification of capable methods is important for both clinical practice and trial research aimed at establishing cholesterol or apoprotein goals. Differences in resistance to detergents may not be the first choice for the detection of LACs, as their characteristics are based on the protein content. Additionally, measuring polypeptides would be insufficient, because the ratio of lipids to proteins was not constant among samples (Fig 2). In fact, the validity of the existing methods for clinical use must be reexamined, because they have been developed to reproduce the results of ultracentrifugation. Immunoaffinity capture is another potential separation method that can be used to prepare certain classes of lipoproteins, as it can handle multiple samples fast and at a low cost. Unfortunately, at present, HDL samples that were isolated using an anti—HDL IgY column contained several nonrelated proteins, such as fibrinogen, immunoglobulins, and albumin [21], suggesting that the affinity of the present system is not sufficiently fine. Of course, the affinity critically depends on the source of “HDL” used in the immunization. Antibodies against single proteins, such as for LACs, would be desirable for the specification of a class of lipoprotein.

**Fig 3:**
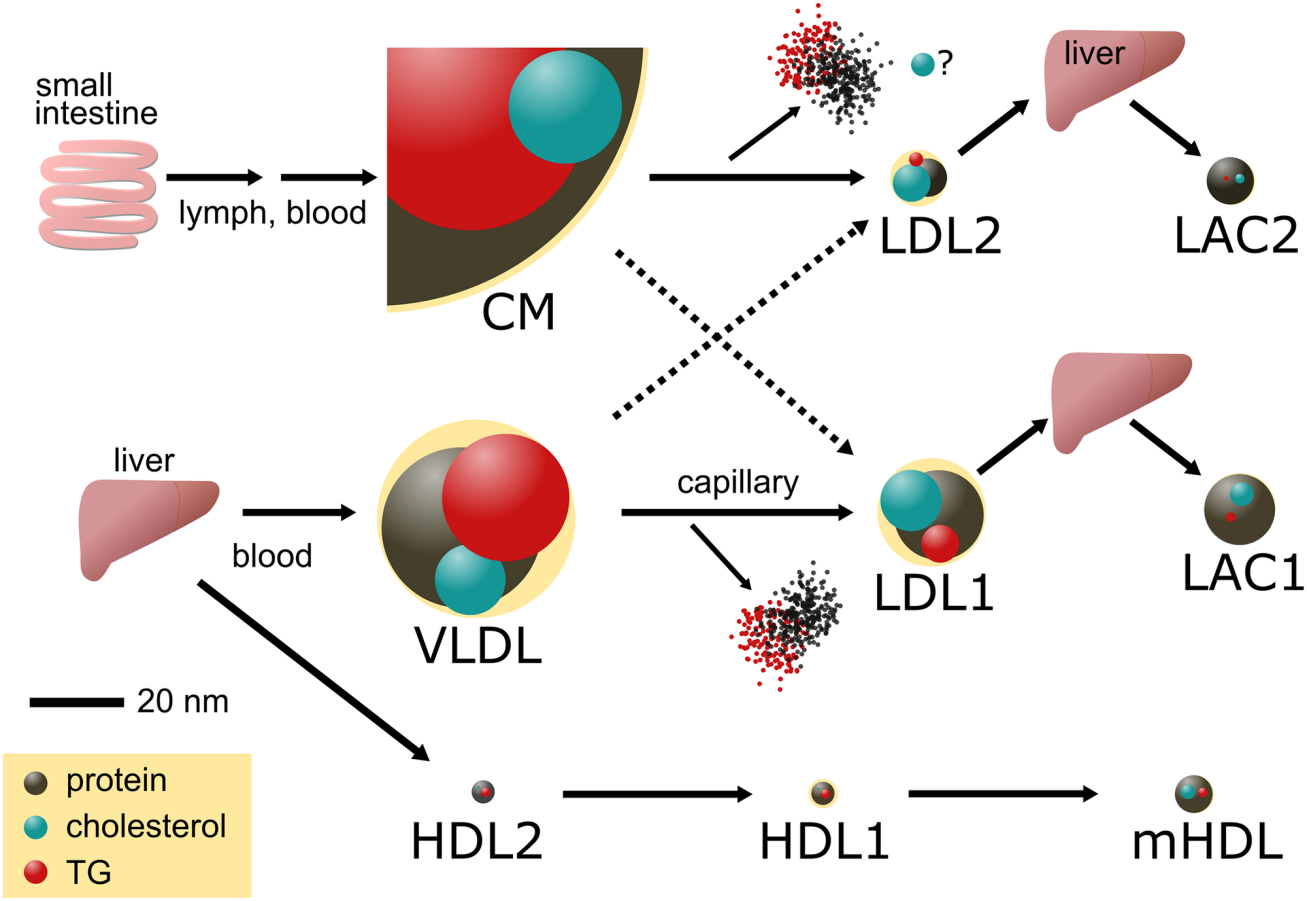
Schematic flowchart of lipoprotein metabolism in rat. A possible pathway is presented indicating the size and composition of the particles. Relative amounts of cholesterol, TG, and proteins are indicated as spheres on a pale-yellow background that indicates the size of the lipoprotein. In the center of the figure, CM and VLDL are degraded to LDL1 or LDL2 by digestion of TG and proteins; some cholesterol would also be removed. The pathways may cross over here because precursors of LDL1 and LDL2 would be particles with ApoB100 and ApoB48, respectively; an ApoB48 polypeptide, if folded in a globular form, would have a size similar to that of the modeled protein included in LDL2.

Both CM and VLDL are stable in serum. A single rare event of digestion transformed these to the smaller LDL; otherwise, the data profiles would be very different (S12 and S13 Figs). In the transformation, a large part of the protein and some cholesterol were also removed from the particles (Table 2, molecules/particle). The mechanisms for removal of these components are unknown. Also, the clear distributions of HDLs suggest that they are stable and that uptake of cholesterol is a rare event. The stability may facilitate some possible pathways of lipoprotein metabolism, although the mechanisms that support several parts are still unknown (Fig 3).

Classes of LDL may contain a much higher level of protein than that thought previously (summarized in Table 2), and the proteins are all unidentified. A predicted function of these proteins is the stabilization of the huge lipidic particles that flow through capillaries via coalescence or dispersion [5]. However, they may also contact specific proteins on the surface of blood vessels. The identification of these proteins and their functions would provide new insights into the circulation and metabolism of lipids. Such proteins could be identified by proteomics studies combined with a proper separation method; the proteins detected previously should be reorganized and classified using a finer system. In fact, the proteins detected in the gel filtration study showed different peak positions [10], which may correspond to the classes found in this study. The identification of the eluted classes may require the use of a curve fitting method of multiple continuous records, such as that presented in this study.

## Conclusions

Lipoproteins consisted of at least 10 different classes that were separated by gel filtration. Those included complexes of LDL and antiproteases. Classes of LDL possessed a much higher level of protein than did those isolated by density gradient centrifugation; some classes of LDL may have densities that are comparable to that of HDLs. Differences in the isolated lipoproteins raise questions pertaining to the validity of density methods, as well as existing analytical methods that are based on it or are being calibrated toward it.

## Supporting information

S1 Fig Relationship between number of distributions and fitting errors. (DOCX)

S2 Fig Relationship of size and elution time of columns used in the HPLC system. (DOCX)

S3 Fig Profiles of lipoproteins. (DOCX)

S4 Fig Logarithmic version of S3 Fig. (DOCX)

S5 Fig Relationship between migration and MW. (DOCX)

S6 Fig Image of PAGE (sample B-D) and quantified protein bands. (DOCX)

S7 Fig Position of fractions and lipoproteins. (DOCX)

S8 Fig Fragment patterns obtained in MALDI-TOF MS. (DOCX)

S9 Fig Slot blot detection of proteins. (DOCX)

S10 Fig Comparison of the measured amount of proteins. (DOCX)

S11 Fig The ratio cholesterol/protein (w/w) in human sample. (DOCX)

S12 Fig Distribution of particles being digested. (DOCX)

S13 Fig Distribution of altered particles. (DOCX)

S14 Fig Size comparisons. (DOCX)

S1 Table Level of lipids and total proteins in the crude serum samples. (DOCX)

S2 Table Level of differences in location and scale parameters estimated using different components. (DOCX)

S3 Table Location parameters found by using different records. (XLSX)

S4 Table Scale parameters found by using different records. (XLSX)

S5 Table Differences between UV records and fitted curves. (DOCX)

S6 Table Parameters found for each sample. (XLSX)

S7 Table Summary of the relationships between lipoprotein and major proteins. (DOCX)

S8 Table Relationship of phospholipid and surface area of the particles, estimated according to Oschry & Eisenberg. (DOCX)

S9 Table Composition assuming that the anti-proteases do not form complexes with LDL. (DOCX)

S1 File Raw HPLC output for sample A. (XLSX)
