# Correction: Lipoproteins comprise at least 10 different classes in rats, each of which contains a unique set of proteins as the primary component

**DOI:** 10.1371/journal.pone.0194258

**Published:** 2018-03-08

**Authors:** Tomokazu Konishi, Yoko Takahashi

Fig 1 is incorrect. The authors have provided a corrected version here.

**Fig 1:**
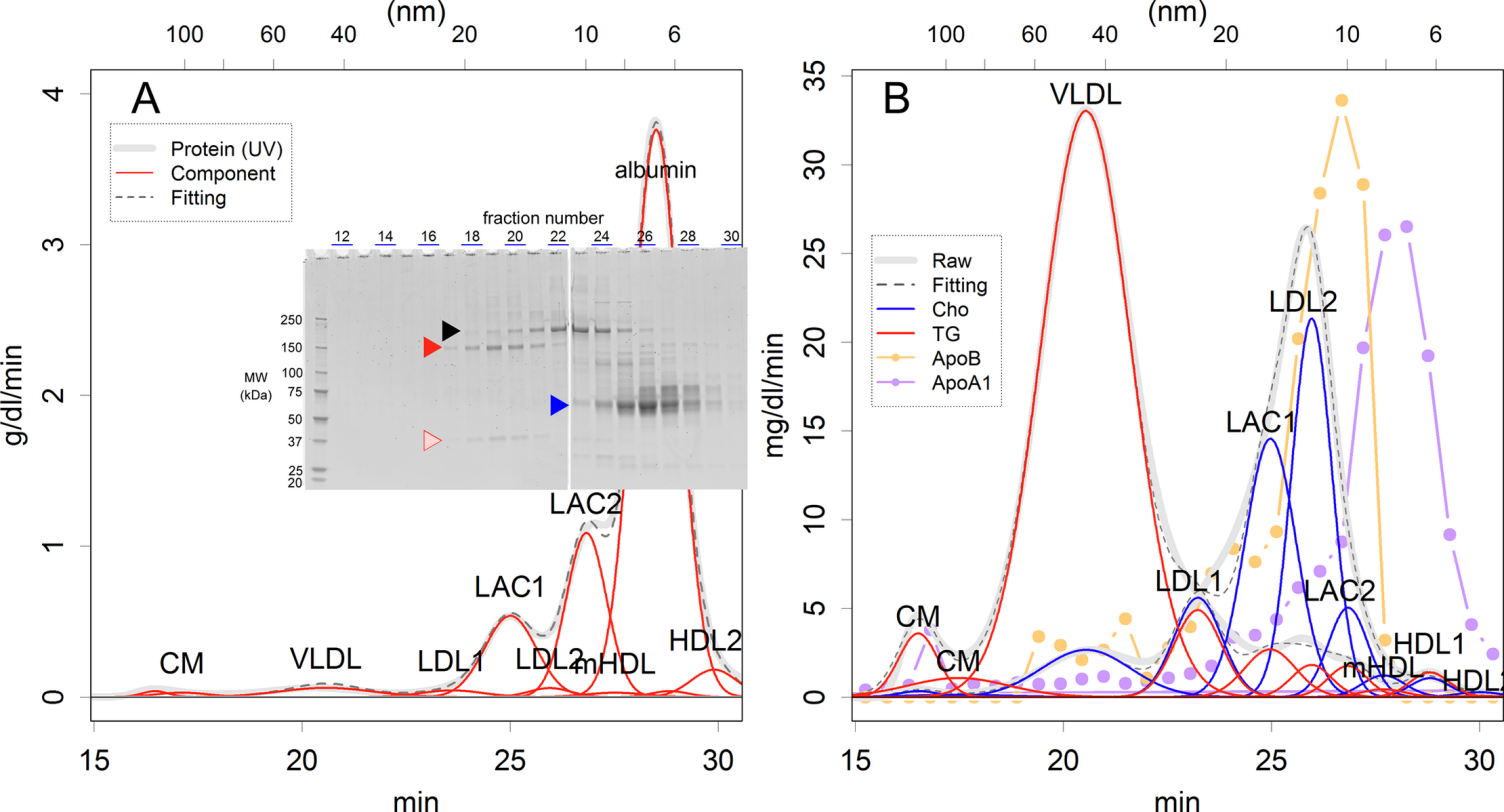
Elution patterns of the gel filtration chromatography. (A) Pattern of UV absorption for monitoring proteins. The positions of fraction numbers on SDS–PAGE analysis correspond to the time on the UV absorption curve. The logarithm of the particle size and the elution time were proportional (S2 Fig). The arrowheads show bands for A1i3 (black), A1m (red and pink), and albumin (blue). (B) Patterns of TG (red) and cholesterol (blue). Evidence from slot blots for ApoA1 and ApoB is also presented (S9 Fig, relative values). Raw: monitoring record; Fitting: sum of the fit curves. Abbreviations for lipoprotein classes are given in the legend of Table 1.
