# Supplementary material for: Lipoproteins comprise at least 10 different classes in rats, each of which contains a unique set of proteins as the primary component
Source: PLoS One. 2018 Feb 20;13(2):e0192955. doi: 10.1371/journal.pone.0192955 (PMC5819787; doi:10.1371/journal.pone.0192955)
Supplement: S1 Fig — (DOCX) [file pone.0192955.s001.docx]

## Relationship between the number of distributions and fitting errors

In general, a greater number of arbitrary parameters can provide better fitting of a model to data. However, the objectivity of the model will be reduced; this becomes obvious in extreme cases. For example, an overfitted model has no falsifiability. In this sense, the number of parameters should be kept as low as possible. Here, the appropriateness of the model used in this manuscript was validated by observing the fitting while reducing and increasing the number of normal distributions that mimic the peaks of classes of lipoproteins.

Fitting of the model to data was evaluated as shown below. For any sample *i*, the difference from the fitted model *M* to data *D* was determined as

$L\left( i \right)=\sum_{t=1}^{n} \left( d_{t,i}-m_{t,i} \right)^{2}$,

where *t* was the time parameter of the record and *n* was the size of data. The expectation of the differences at each recorded point was then estimated as

$R\left( i \right)=\sqrt{{L(i)}/n}$,

and used as an indicator for the fitness.

Whether or not it was necessary to check the number of normal distributions used for fitting, it is obvious that any parameter that covers major components cannot be removed, such as that observed for LDL2 or VLDL. Moreover, for those that have clear biochemical backgrounds, removing the corresponding parameters is pointless. Among the parameters for rather minor ingredients, LAC1 and 2 have not been reported previously. In addition, the second peak of CM may have been the result of an artifact of size exclusion of the column (without the lipoproteins that could not enter into the column beads). Combinations of parameters for these three peaks were removed from the fitting of TG and cholesterol (but not from that of protein, as they cover major protein peaks), to assess the possibility that the remaining parameters would cover the removed ones. In other words, here LAC1 or 2 were estimated to lack lipidic materials. Conversely, another set of parameters was added to the model, for a better fit to VLDL, which would be a mixture of two different biological sources (see the Discussion section). All the fitting processes were carried out automatically under the same conditions used in the manuscript.

A clear tendency toward a finer fitting when using a greater number of parameters was observed (S1 Fig). The level of the worsening of the fit differed among samples, as they contained unique contents of lipoproteins: some of them contained a much greater quantity of LACs than others. The trend toward an improved fitting was clear for 10 parameters, but not 11. Interestingly, the improvements also appeared in protein fitting, which used the full number of parameters; this may have been caused by a decreased precision in the location parameters, which have to recover the removed ones from TG and cholesterol. To fit the various samples used here, at least 10 normal distributions were required; however, additional distributions were not worth considering. The two possible classes of VLDL may have overlapping size distributions, and may not have been well separated by the system used here.


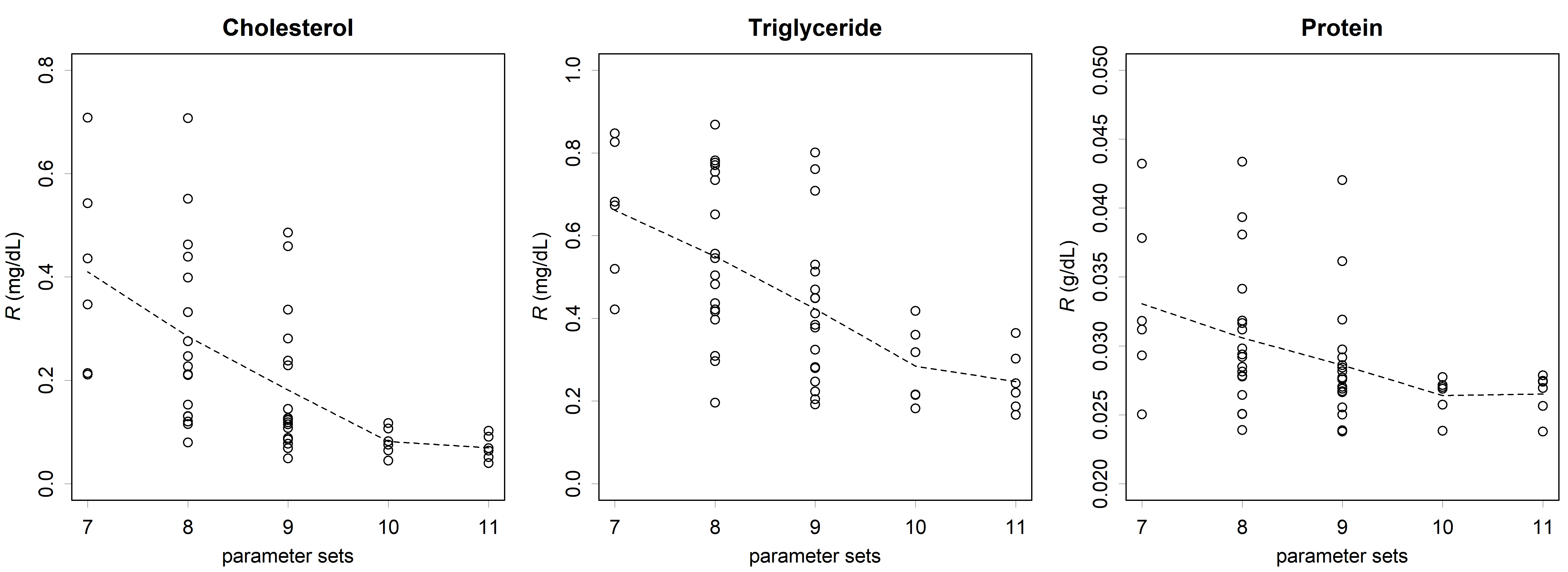


**S1 Fig. Relationship between the number of distributions and fitting errors.** The original number of parameter sets used to fit lipoproteins in the manuscript was 10. They were altered by removing sets of parameters for the second CM, LAC1, and LAC2 from the fitting of TG and cholesterol data, or by adding a new set to cover two classes of VLDL. The dotted lines represent the means of the score *R*.
