# Supplementary material for: Lipoproteins comprise at least 10 different classes in rats, each of which contains a unique set of proteins as the primary component
Source: PLoS One. 2018 Feb 20;13(2):e0192955. doi: 10.1371/journal.pone.0192955 (PMC5819787; doi:10.1371/journal.pone.0192955)
Supplement: S2 Fig — (DOCX) [file pone.0192955.s002.docx]

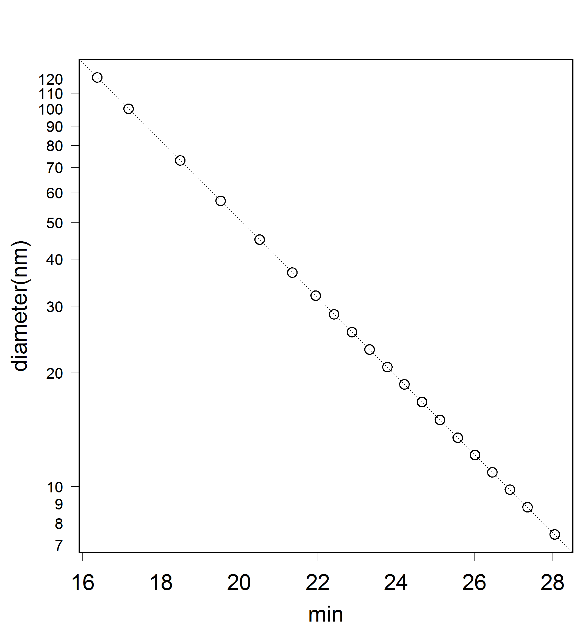


**S2 Fig. Relationship of size and elution time of columns used in the HPLC system.** (provided by Skylight Biotech Inc.) The diameter *d* and elution time *t* correlate as is *d* = 10^ (– 0.1097*t* + 3.872) for the presented units.
