# Supplementary material for: Lipoproteins comprise at least 10 different classes in rats, each of which contains a unique set of proteins as the primary component
Source: PLoS One. 2018 Feb 20;13(2):e0192955. doi: 10.1371/journal.pone.0192955 (PMC5819787; doi:10.1371/journal.pone.0192955)
Supplement: S4 Fig — (DOCX) [file pone.0192955.s004.docx]

**TG and Cho**


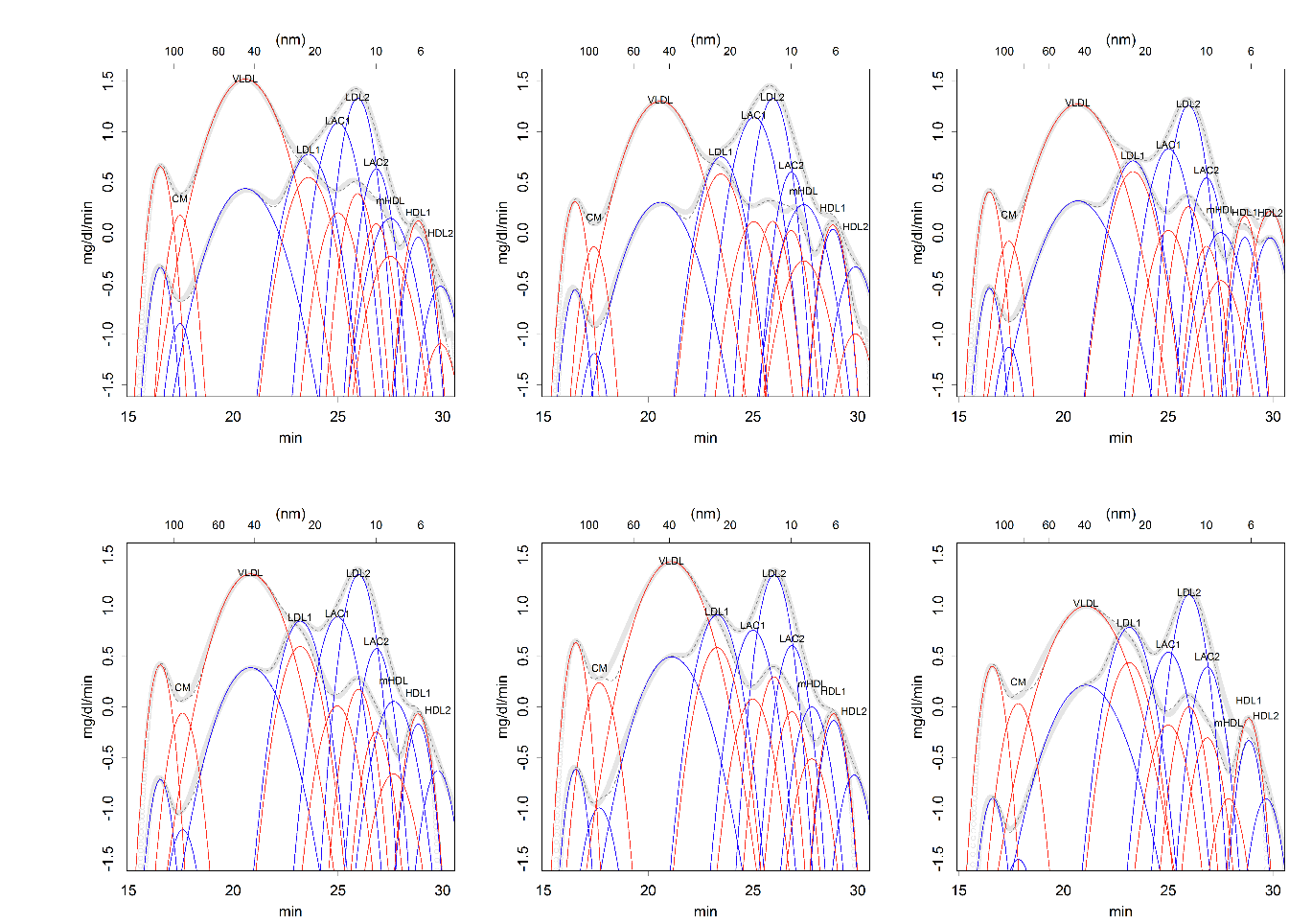


**
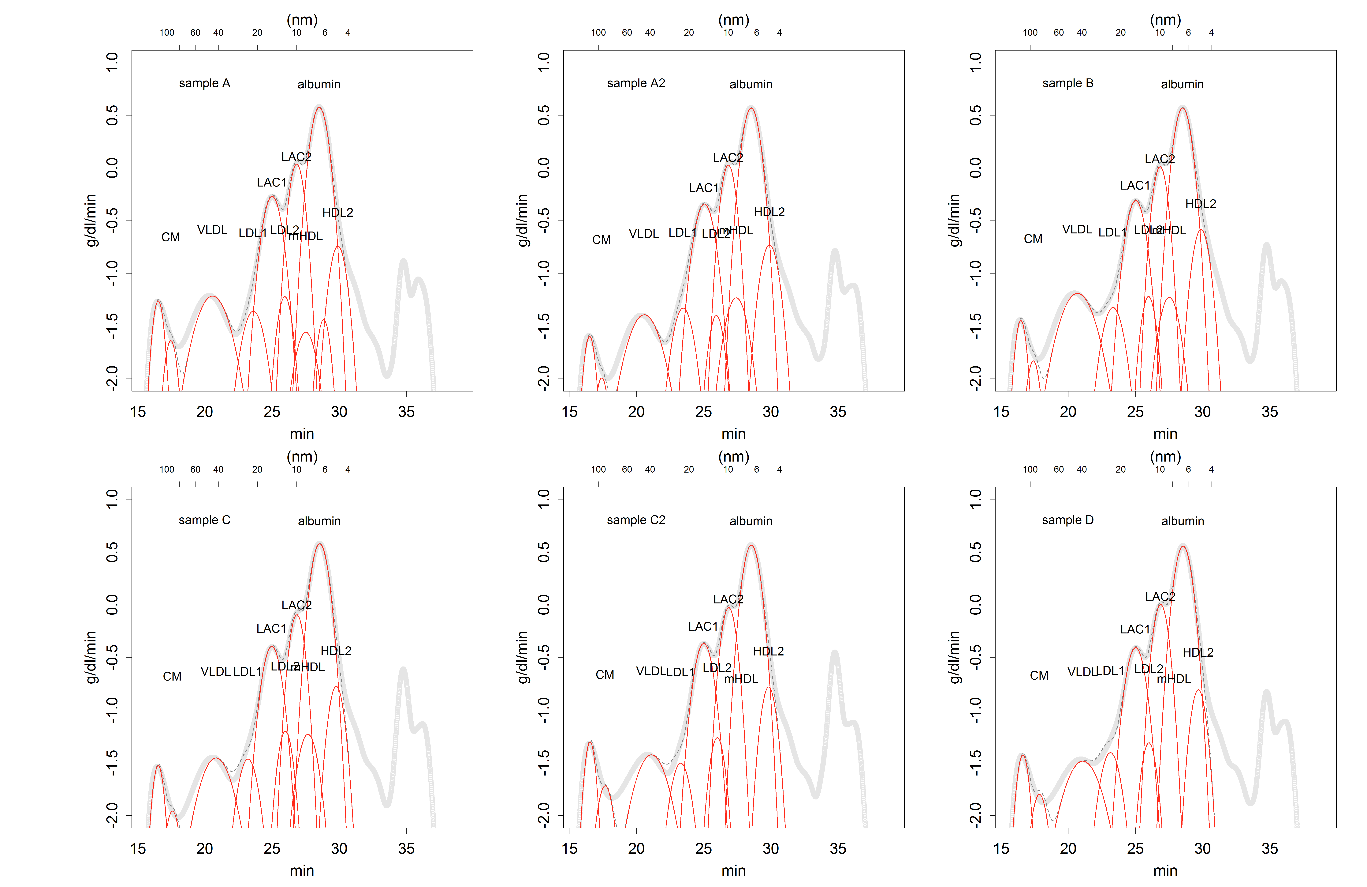
Proteins**

**S4 Fig. Logarithmic version of S3 Fig. TG and Cho:** patterns of TG (red) and cholesterol (blue). **Proteins:** pattern of UV absorption for monitoring proteins. The base of the logarithm is 10; hence, –1 means 0.1 mg/dl/ml, for example. As fittings were performed using real numbers, checking the coincidence to the expanded values by taking logarithms is a strict verification.
