# Supplementary material for: Lipoproteins comprise at least 10 different classes in rats, each of which contains a unique set of proteins as the primary component
Source: PLoS One. 2018 Feb 20;13(2):e0192955. doi: 10.1371/journal.pone.0192955 (PMC5819787; doi:10.1371/journal.pone.0192955)
Supplement: S5 Fig — (DOCX) [file pone.0192955.s005.docx]

## Elution size of proteins detected in SDS–PAGE

Polypeptides detected in SDS–PAGE were quantified and represented in the elution pattern of the gel filtration, and compared with the patterns of lipoproteins.

The proteins were quantified as follows. Proteins were specified by their apparent molecular weight (MW), as estimated by mobility in PAGE (S5 Fig). The images of SDS–PAGE (samples B to D) were obtained as a composite of the signal intensities of the SYPRO stain, which showed coincidence with other protein determinations (S10 Fig). Each of the bands was quantified by scanning the images of PAGE using ImageJ [33]. The intensities of the bands for certain proteins were then normalized; they were divided by their sum. Based on this normalization, the proteins detected in multiple lanes showed a lower and wider shape (S7 Fig). The position and scale of the lipoproteins are reversely indicated from top to bottom.


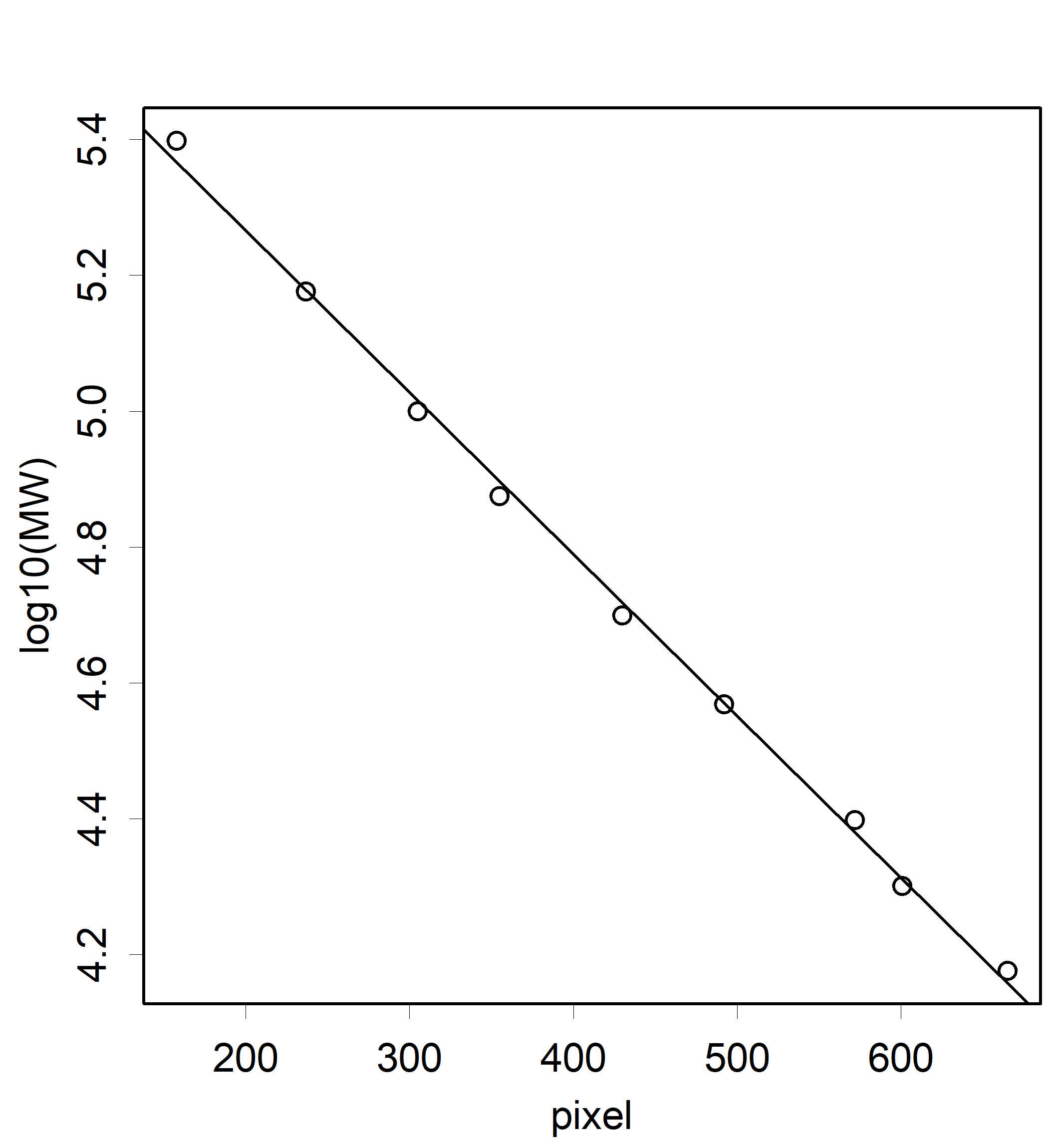
The fractions of major proteins coincided with one or two peaks of related lipoproteins, such as HDL1 and 2, but not LDL and HDL (S7 Table). No major protein was detected without such a relationship to lipoproteins, with the exception of albumin. Albumin was detected by SDS–PAGE and gel filtration, forming the steeper and earlier half of the HDL1 peak.

**S5 Fig. Relationship between migration and MW.**

A linear relationship was observed. The size of each band was estimated accordingly
