# Supplementary material for: Lipoproteins comprise at least 10 different classes in rats, each of which contains a unique set of proteins as the primary component
Source: PLoS One. 2018 Feb 20;13(2):e0192955. doi: 10.1371/journal.pone.0192955 (PMC5819787; doi:10.1371/journal.pone.0192955)
Supplement: S6 Fig — (DOCX) [file pone.0192955.s006.docx]

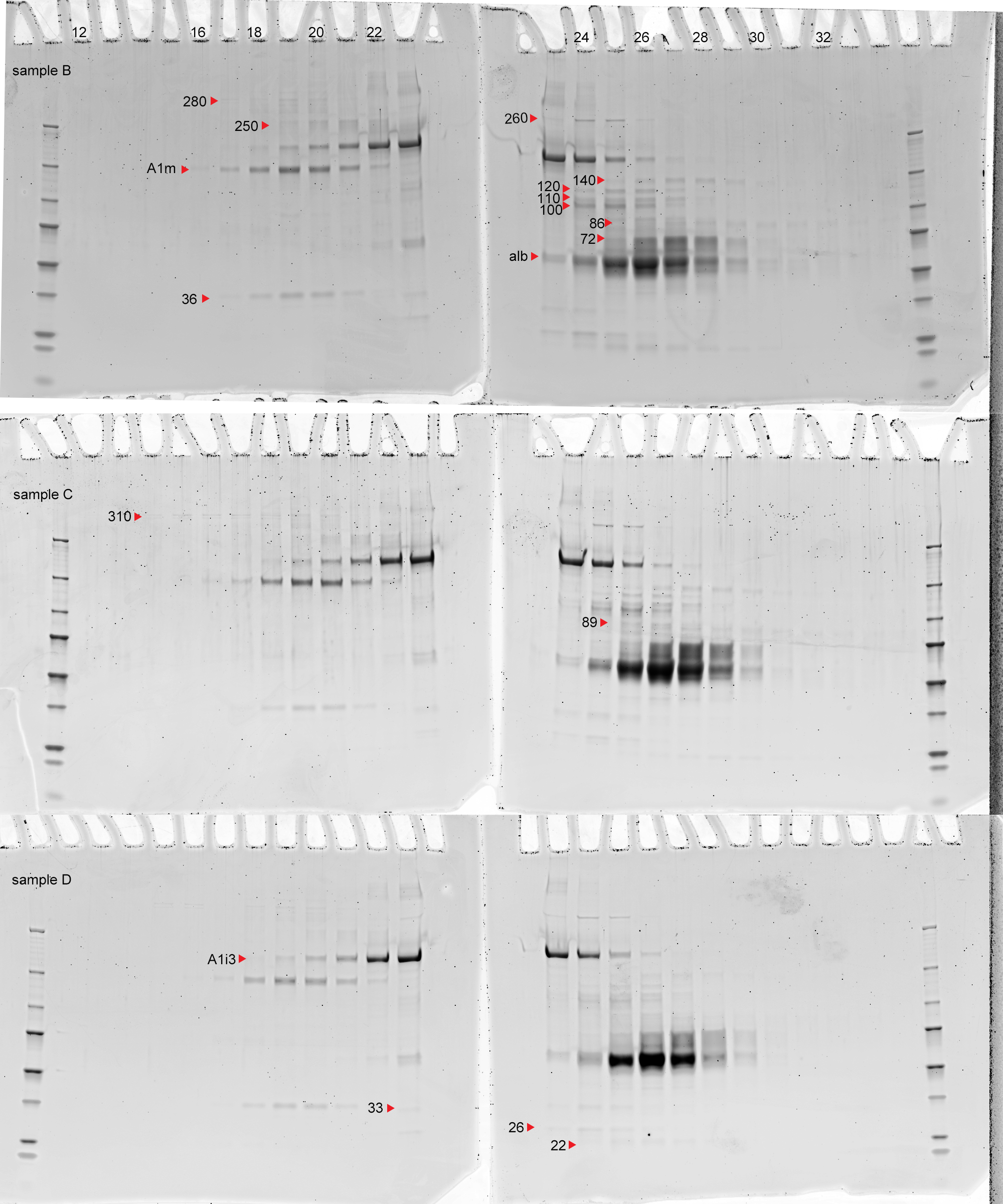


**S6 Fig. Image of PAGE (samples B and D) and quantified protein bands.** The contrast of the images was enhanced for better visibility. The bands used for quantification are indicated. alb, albumin. The numbers indicate the size of polypeptides in kDa.
