# Supplementary material for: Lipoproteins comprise at least 10 different classes in rats, each of which contains a unique set of proteins as the primary component
Source: PLoS One. 2018 Feb 20;13(2):e0192955. doi: 10.1371/journal.pone.0192955 (PMC5819787; doi:10.1371/journal.pone.0192955)
Supplement: S7 Fig — (DOCX) [file pone.0192955.s007.docx]

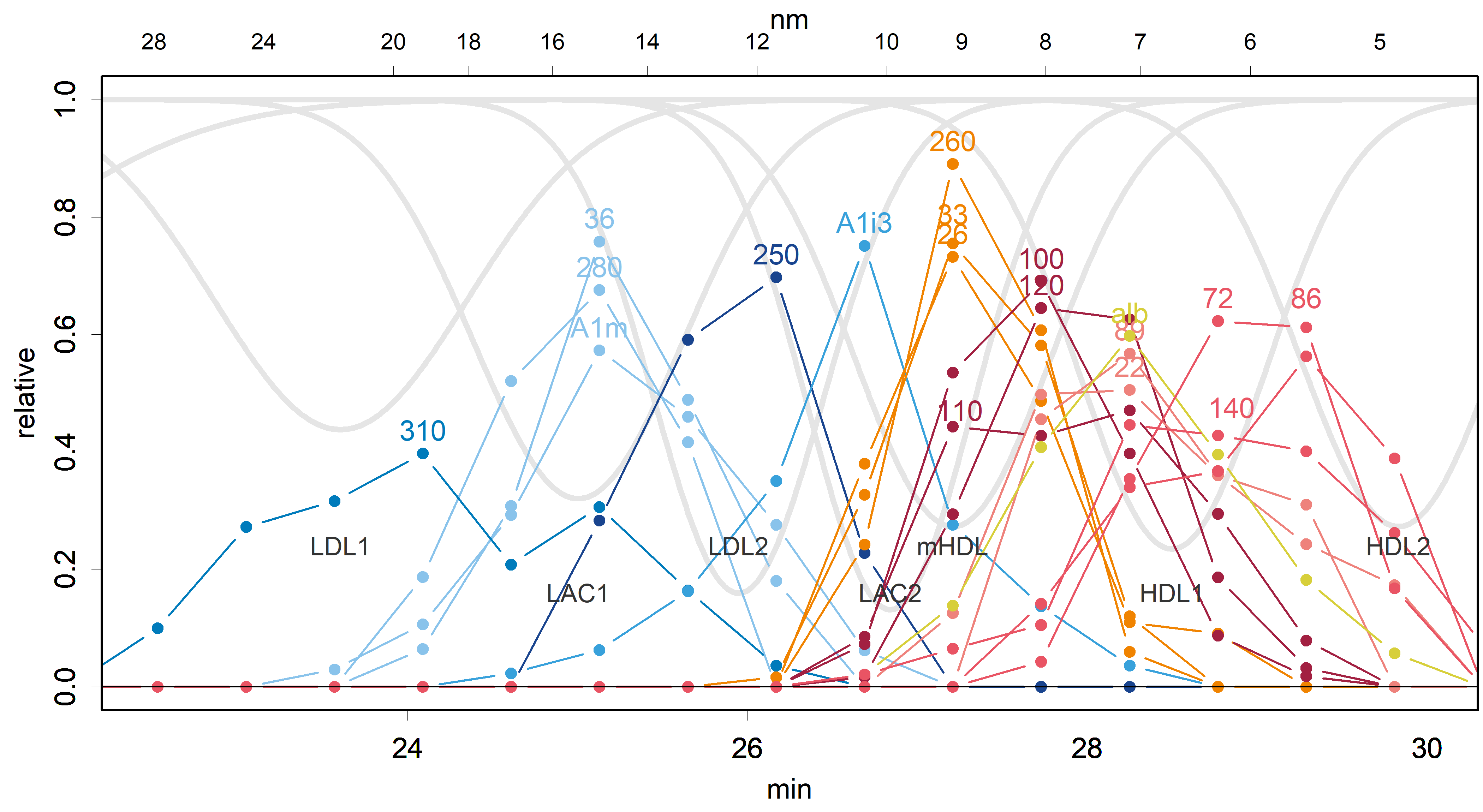


**S7 Fig. Position of fractions and lipoproteins.** The profiles of each protein were taken from one of the four figures, which were selected based on highest signal and lowest noise. The numbers represent the size of polypeptides (kDa).

| class | possible components |
| --- | --- |
| LDL1-LAC1 | 310K |
| LAC1 | 280K, A1m, 36K |
| LDL2 | 250K |
| LAC2 | A1i3 |
| mHDL | 260K, 33K, 26K |
| mHDL-HDL1 | 120K, 110K, 100K |
| HDL1 | 89K, 22K |
| HDL1-HDL2 | 140K, 86K, 72K |

**S7 Table. Summary of the relationships between lipoproteins and major proteins.**
