# Supplementary material for: Lipoproteins comprise at least 10 different classes in rats, each of which contains a unique set of proteins as the primary component
Source: PLoS One. 2018 Feb 20;13(2):e0192955. doi: 10.1371/journal.pone.0192955 (PMC5819787; doi:10.1371/journal.pone.0192955)
Supplement: S8 Fig — (DOCX) [file pone.0192955.s008.docx]

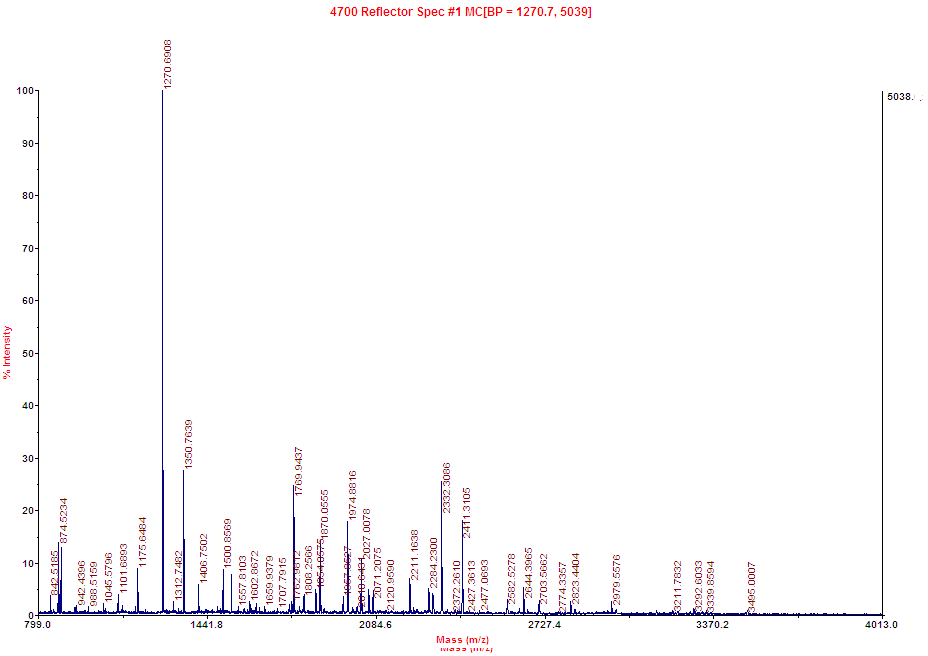


**A**


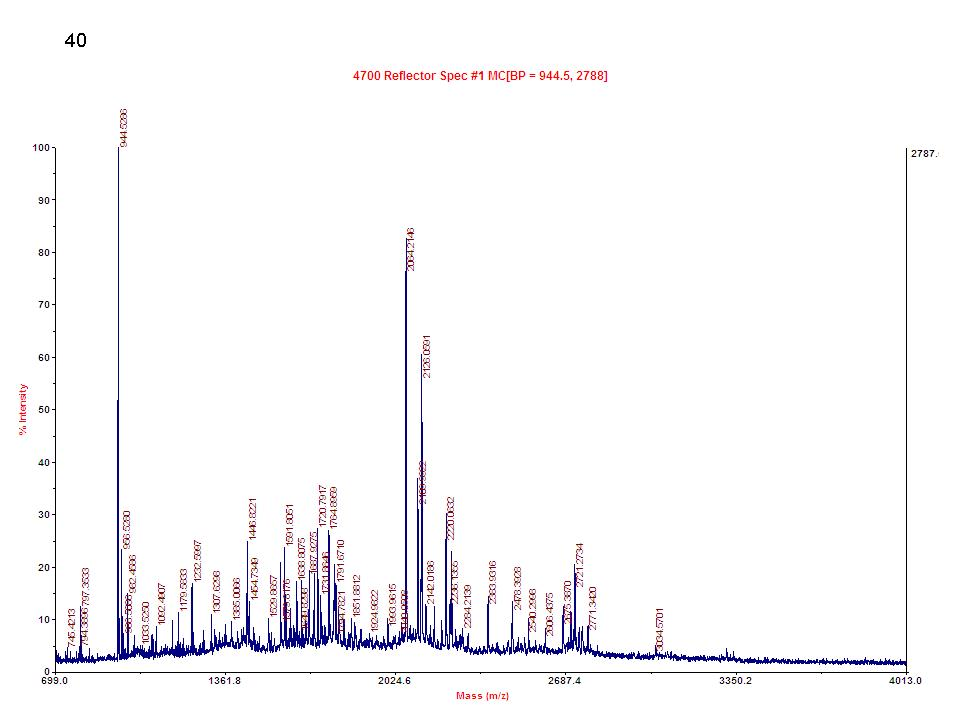


**B**

**S8 Fig.**  **Fragment patterns obtained in MALDI-TOF MS.** (**A**) alpha-1-macroglobulin. (**B**) alpha-1-inhibitor 3. (provided by Genomine Inc.)
