# Supplementary material for: Lipoproteins comprise at least 10 different classes in rats, each of which contains a unique set of proteins as the primary component
Source: PLoS One. 2018 Feb 20;13(2):e0192955. doi: 10.1371/journal.pone.0192955 (PMC5819787; doi:10.1371/journal.pone.0192955)
Supplement: S9 Fig — (DOCX) [file pone.0192955.s009.docx]

## Slot blot analysis of proteins shown in Fig 1B.

**Apo B**
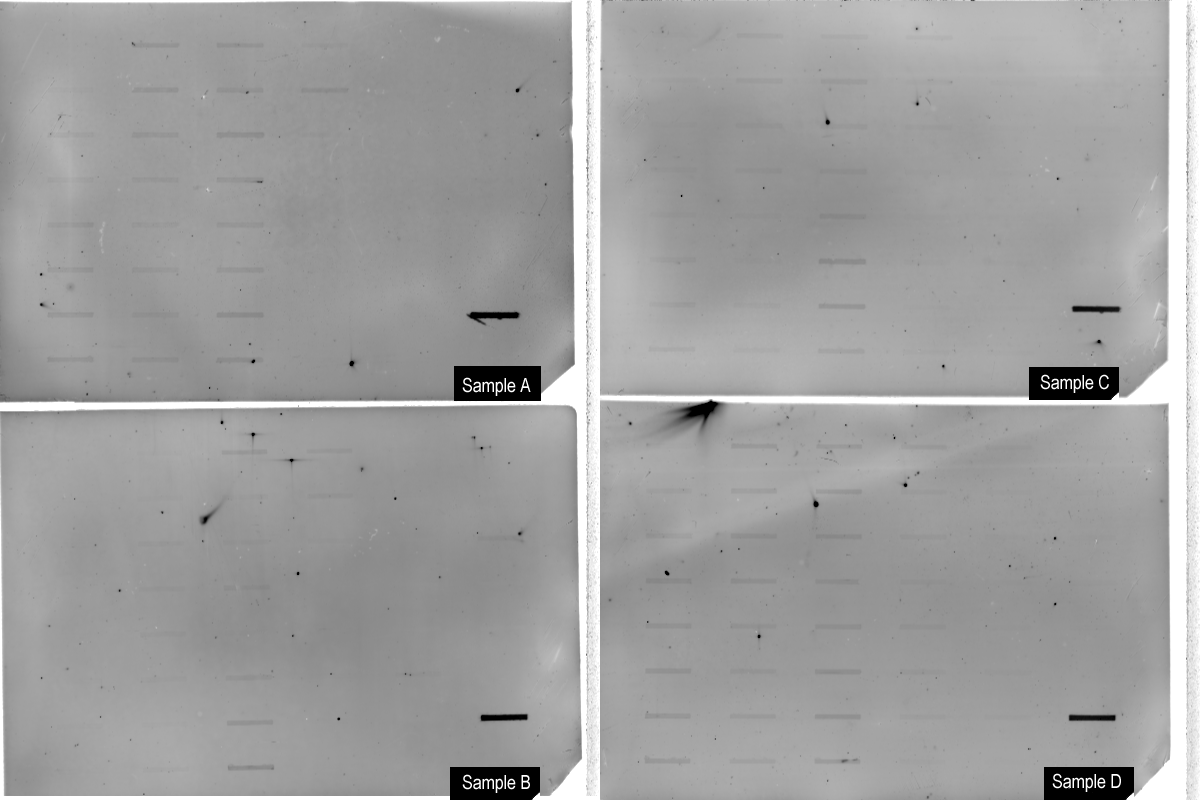


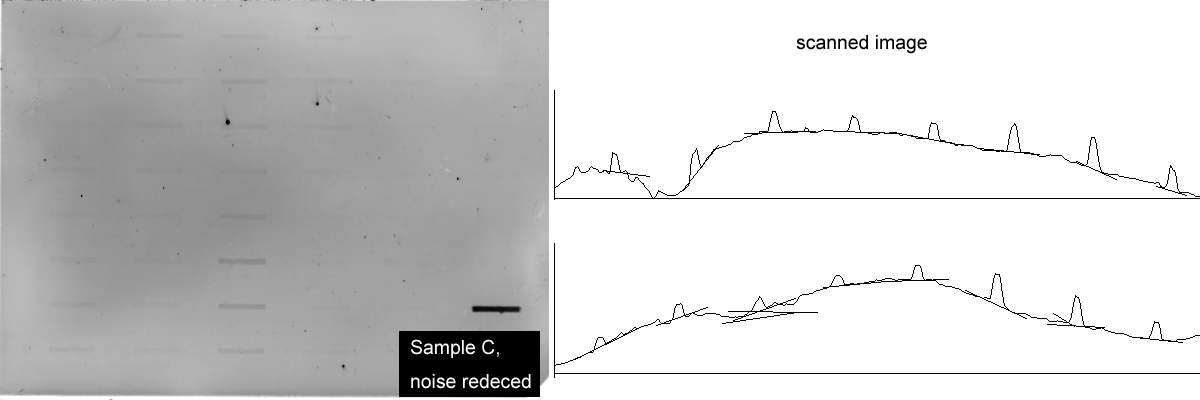


**ApoA**


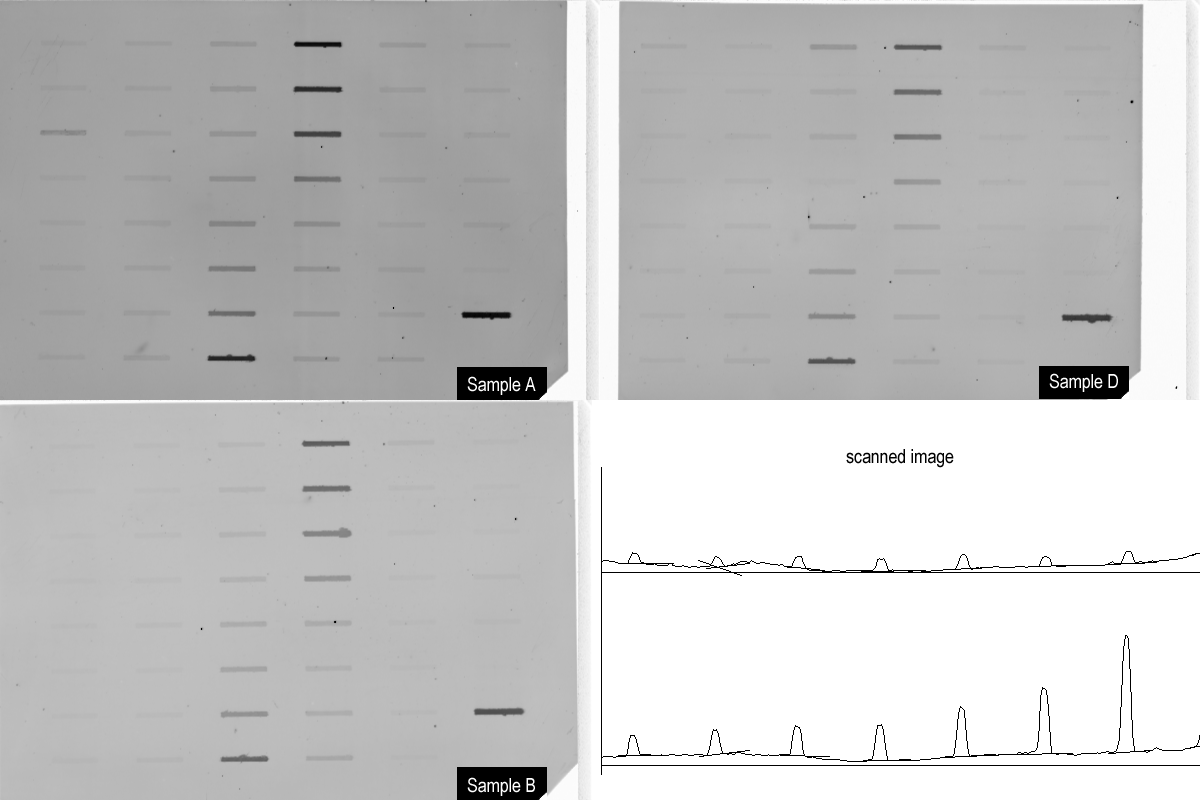


**S9 Fig. Slot blot detection of proteins.** The main components of LDL or HDL, **ApoB** and **ApoA**, are detected in the fractions. The fluorescence on slot membranes was recorded as a raw image (Samples A–D). Small noise spots that may interfere with the quantification of slots were removed by replacing the corresponding pixels with surrounding ones (an example for ApoB in sample C is shown); the images were then scanned from top to bottom. [33] The intensities of signals were measured as peak areas expanded from the background, which inevitably showed a certain level of unevenness (scanned image). The order of the fractions is (from the left-most column and from top to bottom): 1–8, 9–16, 17–24, etc. Crude serum was placed in the 47th slot, as a positive control.
