# Supplementary material for: Lipoproteins comprise at least 10 different classes in rats, each of which contains a unique set of proteins as the primary component
Source: PLoS One. 2018 Feb 20;13(2):e0192955. doi: 10.1371/journal.pone.0192955 (PMC5819787; doi:10.1371/journal.pone.0192955)
Supplement: S10 Fig — (DOCX) [file pone.0192955.s010.docx]

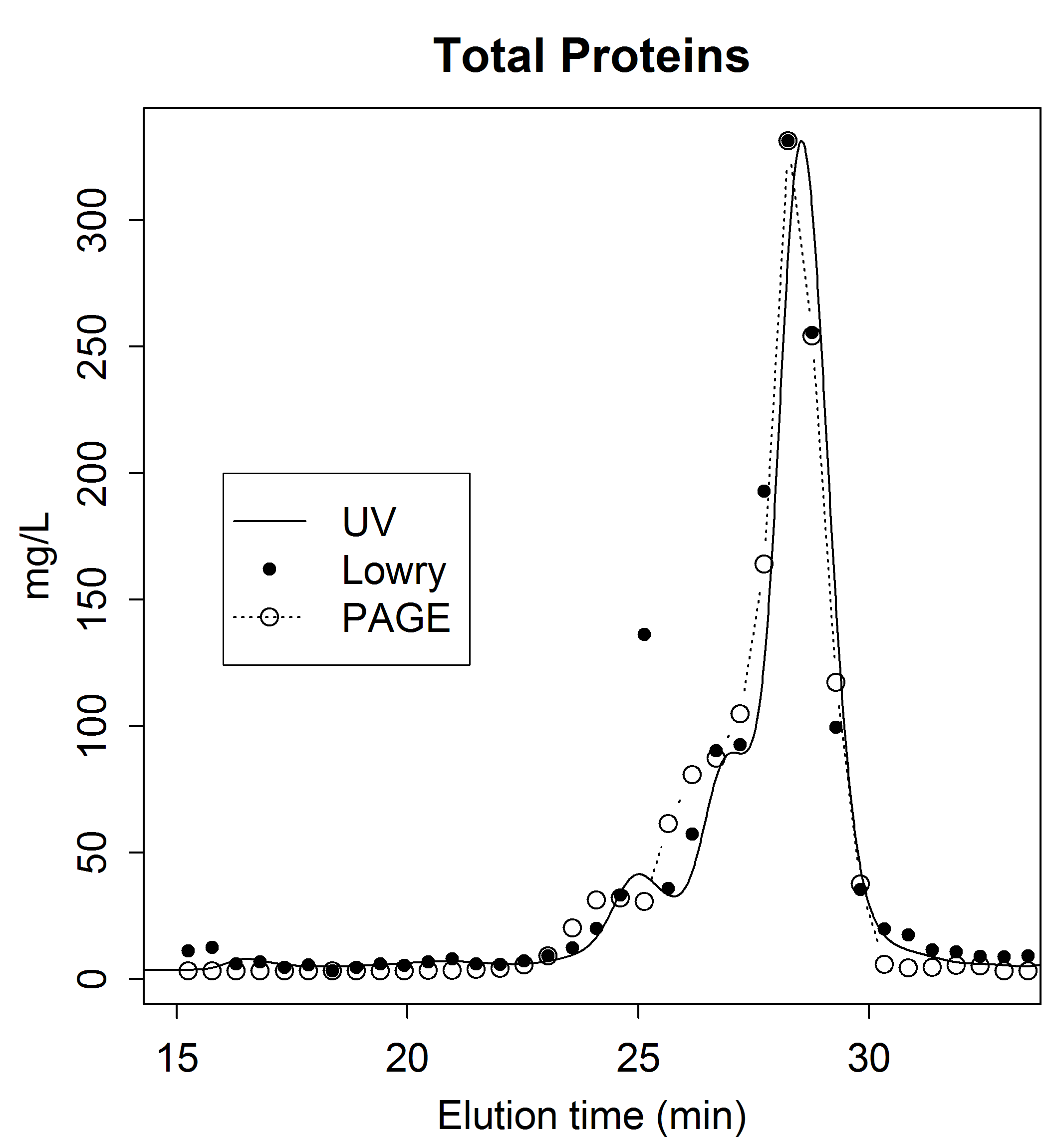


**S10 Fig. Comparison of the measured amount of proteins.** The concentration of total protein in the fractionated sample was performed using the Lowry method. Protein bands were detected of SDS–PAGE and measured using fluorescence. Elution was continuously monitored for UV absorption. The signal strength of both fluorescence and UV was scaled to fit the results of the Lowry method.
