# Supplementary figures and images for: Lipoproteins comprise at least 10 different classes in rats, each of which contains a unique set of proteins as the primary component
Source: PLoS One. 2018 Feb 20;13(2):e0192955. doi: 10.1371/journal.pone.0192955 (PMC5819787; doi:10.1371/journal.pone.0192955)

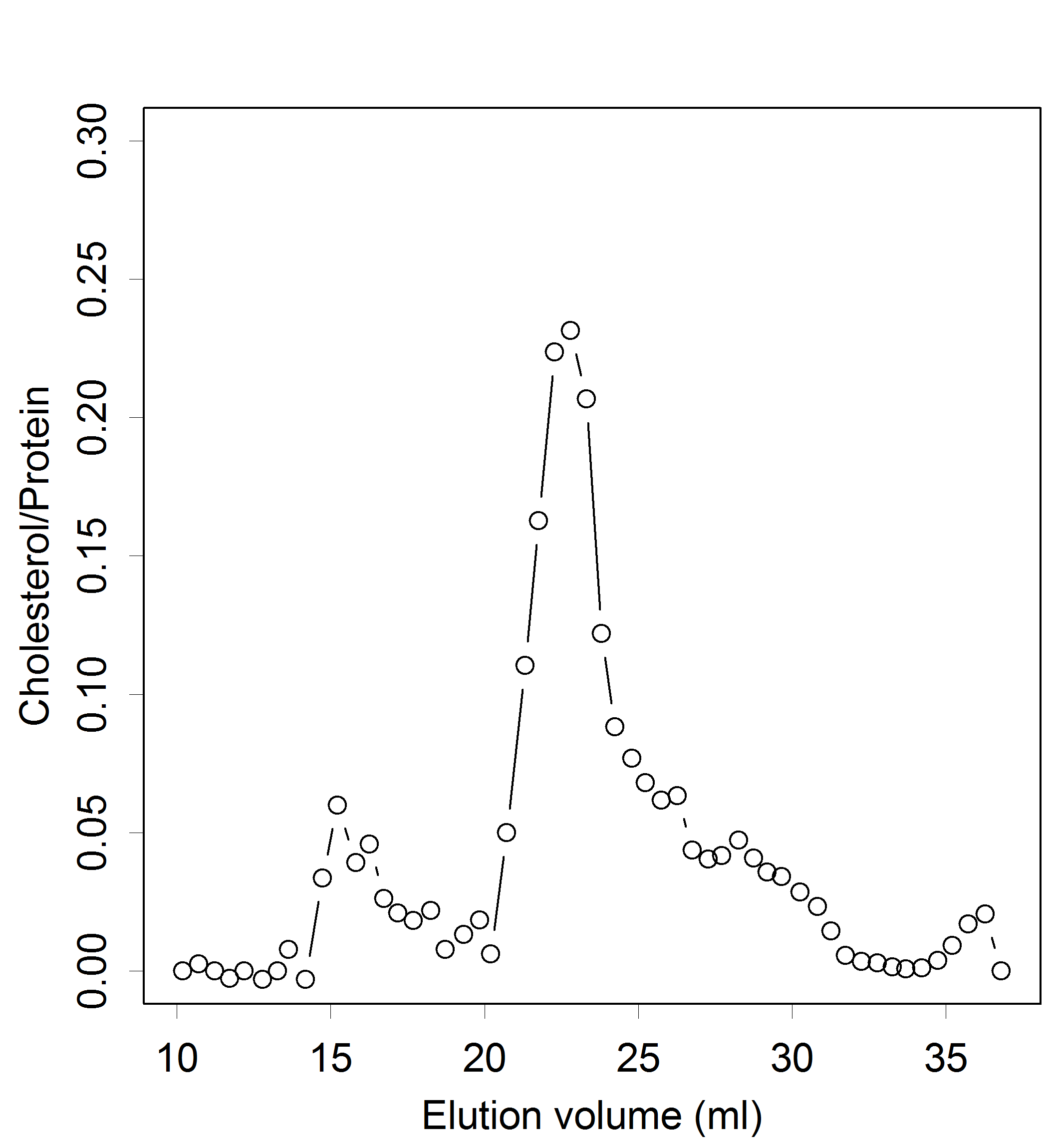


**S11 Fig. The ratio cholesterol/protein (w/w) in human sample**. Ratios were calculated from Gordon et al. (2010).[9]

Supplement: S11 Fig — (DOCX) [file pone.0192955.s011.docx]
