# Supplementary material for: Lipoproteins comprise at least 10 different classes in rats, each of which contains a unique set of proteins as the primary component
Source: PLoS One. 2018 Feb 20;13(2):e0192955. doi: 10.1371/journal.pone.0192955 (PMC5819787; doi:10.1371/journal.pone.0192955)
Supplement: S12 Fig — (DOCX) [file pone.0192955.s012.docx]

# Data simulation

## If LDL is gradually degraded by lipoprotein lipase (LPL) like a melting snowman

This analysis predicts the profile of TG if LDL is repeatedly degraded in serum. It assumes that just after being released to serum, TG is removed from LDL with a certain frequency that may be proportional to the concentration of LDL in serum regardless of the size of the particle and that all LDL contains a single ApoB apoprotein, which interacts with a specific receptor that is connected to LPL. Hence, the frequency of TG removal is determined by the concentration of ApoB protein. We also assumed that LDL is continuously supplied into serum at a constant rate, that particles that become smaller than a threshold value are removed from the system, and that the level of LDL is maintained by a balance of supply and degradation, maintaining a steady state. The size of the supplied LDL is presumed to be log-normally distributed, showing a peak on the gel permeation column with a time of 17 and a range of 0.48 (min) of normal distribution.

In simulating the degradation, we set a very short period. Within that period, 0.2% of the released particles were degraded, altering the distribution. In the next moment, the altered distribution would be further altered. We tested two possible determinants of degradation velocity: the particle volume and the enzyme activity.

If degradation velocity is determined by the volume of the particle, each degradation will remove a certain proportion of TG: the bigger the particle, the more TG will be removed. Repeating this process would expand the time and range parameters (S12A Fig). Because the simulated system is in a steady state, in any moment, it includes all the conditions for particles being digested, from those just released to those that have existed longer in the system, with the same probability. Therefore, the distribution of the system can be estimated by integration of the simulated distributions, and the integrated distribution is uniform between release and removal (S12B Fig).

**
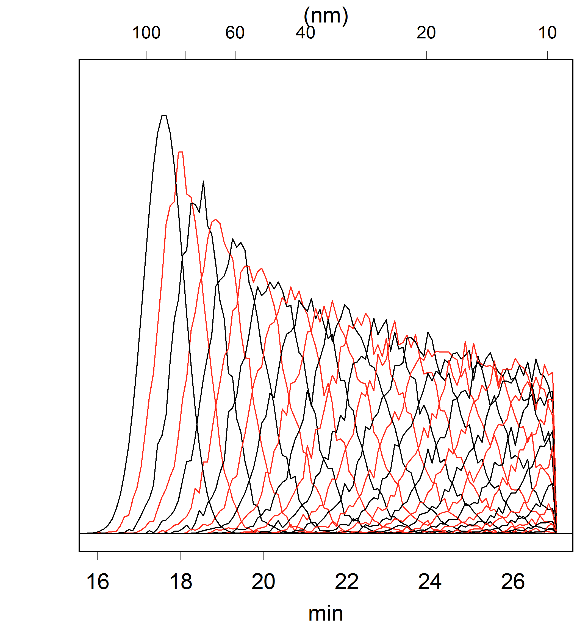

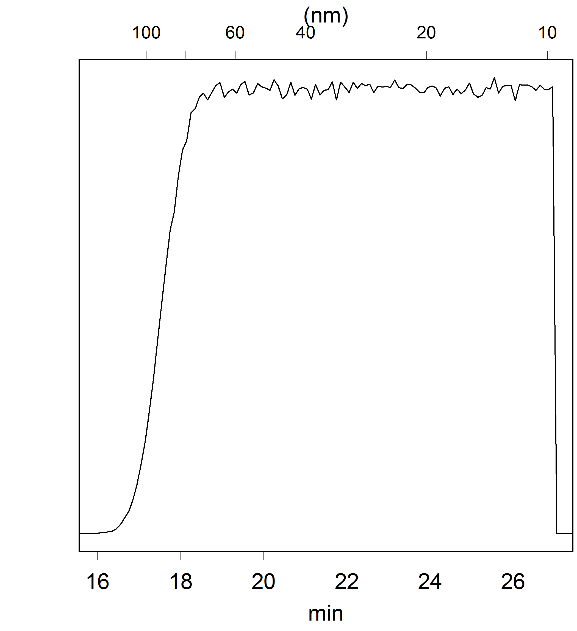
**

**A B**

**S12 Fig. Distribution of particles being digested.** (**A**) Supplied particles (left end) are degraded, losing 10% of their volume. The distributions of every 1,500 trials are layered and presented in black and red alternately. The y-axis shows relative value of TG. **(B)** Expected distribution of the system: a fixed proportion of TG is removed from all particles. Those for each trial were summed, estimating a steady state.
