# Supplementary material for: Lipoproteins comprise at least 10 different classes in rats, each of which contains a unique set of proteins as the primary component
Source: PLoS One. 2018 Feb 20;13(2):e0192955. doi: 10.1371/journal.pone.0192955 (PMC5819787; doi:10.1371/journal.pone.0192955)
Supplement: S13 Fig — (DOCX) [file pone.0192955.s013.docx]

# Data simulation (cont.)

**
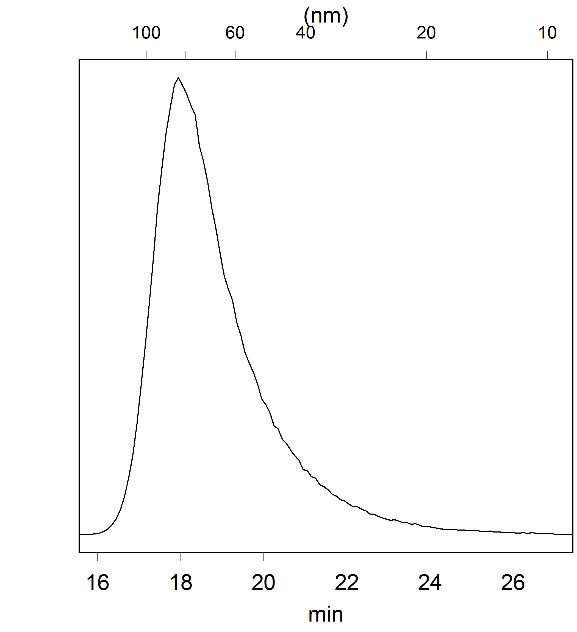

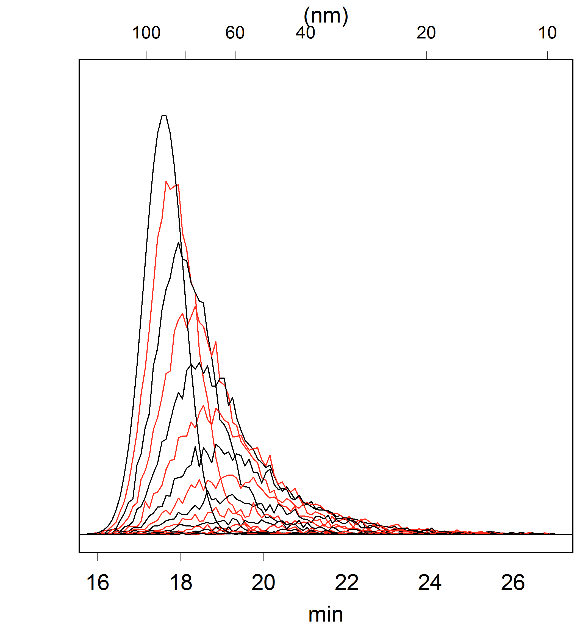
**

**A B**

**S13 Fig. Distribution of altered particles.** (**A**) The supplied particles (left end) were degraded, losing a constant volume of 2.7% of the median of that of the supplied particle. The distributions of every 3,000 trials are presented. **(B)** Expected distribution of the system: a fixed volume is removed from all particles.

If degradation is determined by the activity of the LPL enzyme, each degradation would remove a certain volume of TG, regardless of the size of the particle. Repeating this process reduces the size distributions more rapidly (S13A Fig), and the integrated distribution is heavily skewed (S13B Fig).

Neither of the simulations represented the clearly normal distribution patterns of the serum profiles. Therefore, the classes of lipoproteins seen in the profile are fairly stable; a single degradation may remove most of the TG available, changing each class of LDL to the next.
