# Supplementary material for: Lipoproteins comprise at least 10 different classes in rats, each of which contains a unique set of proteins as the primary component
Source: PLoS One. 2018 Feb 20;13(2):e0192955. doi: 10.1371/journal.pone.0192955 (PMC5819787; doi:10.1371/journal.pone.0192955)
Supplement: S14 Fig — (DOCX) [file pone.0192955.s014.docx]

## Estimation of forces in the density gradient centrifugation

During the centrifugation process, samples were treated under extremely high gravity. Particles dispersed in a dense medium gain both weight and buoyancy according to the gravity. They will migrate at a constant velocity in the fluid, as the force of viscosity occurs in balance with the weight and buoyancy. The forces may be sufficient to deconstruct certain binding events among materials, as follows.

Let an LDL particle (density, 1.01E3 kg/m^3^; diameter, *d* = 100 nm) in dense fluid (density, 1.2 E3 kg/m^3^) be placed under gravity of 5E5 g. The volume and mass of the particle are *V*_l_ = π ⁄ 6*d*^3^ = 5.2E–22 m^3^ and *m* = *V*_l_ *1.01E3 = 5.2E–19 kg, respectively. The weight is *G* = *m**5E5*9.8 = 2.6E–12 N and the buoyancy is *F*_l_ = *V*_l_*1.2E3*5E5*9.8 = 3.1E-12 N. As *F*_l_ > *G*, the particle is pulled upward.

Then, let a globule protein complex of the same mass be attached to the LDL particle via a hydrophobic effect (density of the protein, 1.43E3 kg/m^3^). If the volume of the protein is *V*_p_ = *m*/1.43E3 = 3.7E–22 m^3^, then the buoyancy is *F*_p_ = *V*_p_*1.2*5E5*9.8 = 2.2E–12 N. As *F*_p_ < *G*, the protein is pulled downward. Therefore, the LDL–protein complex receives tensile force of *F*_l_ – G – (*F*_p_ – G) = 0.9 nN.

How large should this force be for a lipidic molecule? The hydrophobic effect between single layers of lipid and/or peptides were measured using AFM, which showed 0.05–5 nN of pull-off force. [40] By estimating the contacting area of the AFM tip that was coated by a 2 nm layer of titanium, 20 nm of gold, and 2 nm of monolayer lipid, 10–100 nm^2^, the adhesion stress would be similar to the LDL–protein complex studied here. Therefore, the gravity force could be sufficiently large, as hydrophobic effects are derived by the entropic effect of bindings among water molecules inside the lipidic particle, which water molecules cannot access, and the effects will be weakened further. Hence, pulling proteins into one side of a lipoprotein would require a smaller force, which may produce the subjected condition (half of a molecule is proteinous and the rest is lipidic).

Once the complex collapses into proteinous and lipidic parts, these will sink and float separately, gaining certain viscosity forces that balance with the weight and buoyancy, thus giving shearing force to the particles. In contrast, among particles with similar densities, the hydrophobic effect will facilitate binding and remixing, to form larger particles. Those shearing and binding forces will achieve artificial states of lipoproteins with better stability under high-gravity conditions.


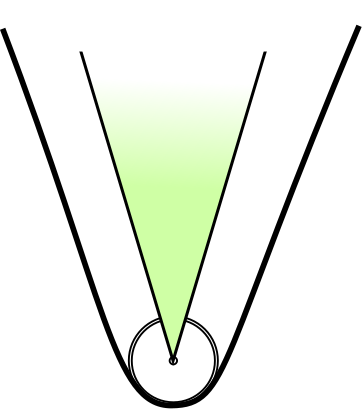


AFM tip


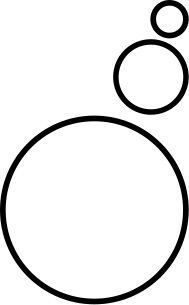


20 nm

40 nm

100 nm

coated tip

**S14 Fig. Size Comparisons. Left:** edge of AFM tip, coated by lipid layer. **Right:** particles with indicated diameters.
