# Supplementary material for: Lipoproteins comprise at least 10 different classes in rats, each of which contains a unique set of proteins as the primary component
Source: PLoS One. 2018 Feb 20;13(2):e0192955. doi: 10.1371/journal.pone.0192955 (PMC5819787; doi:10.1371/journal.pone.0192955)
Supplement: S1 Table — (DOCX) [file pone.0192955.s015.docx]

|  | PL (g/L) | TG (g/L) | cholesterol (g/L) | protein (g/L) |
| --- | --- | --- | --- | --- |
| Sample A | 1.636 | 0.598 | 0.768 | 73.8 |
| Sample A2 | 1.696 | 0.461 | 0.770 | 84.1 |
| Sample B | 1.379 | 0.744 | 0.571 | 80.7 |
| Sample C | 1.301 | 0.704 | 0.632 | 75.5 |
| Sample C2 | 1.346 | 0.622 | 0.671 | 76.9 |
| Sample D | 0.845 | 0.389 | 0.347 | 73.6 |

**S1 Table. Level of lipids and total proteins in the crude serum samples.** The clear differences in the levels of lipids and proteins would be the consequence of feedings to the animals (Materials and Methods).
