# Supplementary material for: Lipoproteins comprise at least 10 different classes in rats, each of which contains a unique set of proteins as the primary component
Source: PLoS One. 2018 Feb 20;13(2):e0192955. doi: 10.1371/journal.pone.0192955 (PMC5819787; doi:10.1371/journal.pone.0192955)
Supplement: S2 Table — (DOCX) [file pone.0192955.s016.docx]

## Location and scale parameters found in different components

|  | Profile | Time | Range |
| --- | --- | --- | --- |
| CM(v) | TG | 0.04 | 0.02 |
| CM | TG | 0.20 | 0.13 |
| VLDL | TG | 0.19 | 0.08 |
| LDL1 | Cho | 0.07 | 0.04 |
| LAC1 | Protein | 0.10 | 0.03 |
| LDL2 | Cho | 0.03 | 0.01 |
| LAC2 | Protein | 0.07 | 0.02 |
| mHDL | Cho | 0.09 | 0.13 |

**S2 Table.** **Level of differences in location and scale parameters estimated using different components.** The parameters were first estimated using the components indicated in the profile column and then re-estimated for the other components. The levels of difference were evaluated by the variance for each sample. The square root of the mean of the variances is shown in the location and scale columns (min), indicating the average noise level used in estimating these parameters.
