# Supplementary material for: Lipoproteins comprise at least 10 different classes in rats, each of which contains a unique set of proteins as the primary component
Source: PLoS One. 2018 Feb 20;13(2):e0192955. doi: 10.1371/journal.pone.0192955 (PMC5819787; doi:10.1371/journal.pone.0192955)
Supplement: S5 Table — (DOCX) [file pone.0192955.s019.docx]

| sample | A | A2 | B | C | C2 | D |
| --- | --- | --- | --- | --- | --- | --- |
| difference (%) | 3.06 | 2.92 | 2.89 | 2.72 | 2.75 | 2.90 |

**S5 Table. Differences between UV records and fitted curves.** Difference *d* was calculated as

$d= \left( \frac{\sum\left| r_{t}-c_{t} \right|}{\sum r_{t}} \right)\times100$,

where *r* is the record and *c* is the fitted curve at every recorded time point *t* (15–30 min of elution). It should be noted that *d* includes both the gaps of fittings and the noise of records.

The diameter *d* and the elution time *t* correlate as *d* = 10^ (–0.1097*t* + 3.872) for the presented units.
