# Supplementary material for: Lipoproteins comprise at least 10 different classes in rats, each of which contains a unique set of proteins as the primary component
Source: PLoS One. 2018 Feb 20;13(2):e0192955. doi: 10.1371/journal.pone.0192955 (PMC5819787; doi:10.1371/journal.pone.0192955)
Supplement: S7 Table — (DOCX) [file pone.0192955.s021.docx]

| class | possible components |
| --- | --- |
| LDL1-LAC1 | 310K |
| LAC1 | 280K, A1m, 36K |
| LDL2 | 250K |
| LAC2 | A1i3 |
| mHDL | 260K, 33K, 26K |
| mHDL-HDL1 | 120K, 110K, 100K |
| HDL1 | 89K, 22K |
| HDL1-HDL2 | 140K, 86K, 72K |

**S7 Table. Summary of the relationships between lipoproteins and major proteins.**
