# Supplementary material for: Lipoproteins comprise at least 10 different classes in rats, each of which contains a unique set of proteins as the primary component
Source: PLoS One. 2018 Feb 20;13(2):e0192955. doi: 10.1371/journal.pone.0192955 (PMC5819787; doi:10.1371/journal.pone.0192955)
Supplement: S8 Table — (DOCX) [file pone.0192955.s022.docx]

|  |  | | Model | | | | |
| --- | --- | --- | --- | --- | --- | --- | --- |
|  |  | S/V | A | B | C | D | E |
| VLDL | Non-fasted | 1.39E+08 | 8.6806 | –0.524 | 8.612 | 8.717 | 8.717 |
|  | Fasted |  | 8.7325 | –0.562 | 8.675 | 8.749 | 8.762 |
| LDL | Non-fasted | 2.74E+08 | 8.4208 | 0.059 | 8.657 | 8.794 | 8.762 |
|  | Fasted |  | 8.3973 | 0.015 | 8.640 | 8.762 | 8.727 |
| HDL1 | Non-fasted | 4.72E+08 | 7.9616 | –0.142 | 8.576 | 8.672 | 8.635 |
|  | Fasted |  | 7.766 | –0.137 | 8.588 | 8.672 | 8.605 |
| HDL2 | Non-fasted | 6.52E+08 | 7.8128 | –0.156 | 8.784 | 8.838 | 8.717 |
|  | Fasted |  | 7.4448 | –0.182 | 8.750 | 8.797 | 8.660 |
|  |  | SD | 0.4705 | 0.226 | 0.074 | 0.060 | 0.058 |
|  |  | Mean | 8.1521 | –0.204 | 8.660 | 8.750 | 8.698 |
|  |  | SD/Mean | 0.0577 | –1.112 | 0.0086 | 0.0069 | 0.0067 |

**S8 Table. Relationship of phospholipid and surface area of the particles, estimated according to Oschry & Eisenberg.** [7] The ratio of surface and volume (S/V) was derived from electron microscopic images. The relationships were estimated from the following expressions: A: CE/PL*S/V; B: Cho/PL*S/V; C: (TG+CE)/(UC+PL)*S/V; D:(TG+CL)/PL*S/V; E: (TG+Cho)/PL*S/V. The stability of the values was defined as the SD of the logarithmic values divided by the means. The composition of phospholipid used in Table 2 was estimated using model E.
