# Supplementary material for: Lipoproteins comprise at least 10 different classes in rats, each of which contains a unique set of proteins as the primary component
Source: PLoS One. 2018 Feb 20;13(2):e0192955. doi: 10.1371/journal.pone.0192955 (PMC5819787; doi:10.1371/journal.pone.0192955)
Supplement: S9 Table — (DOCX) [file pone.0192955.s023.docx]

|  | CM | VLDL | LDL1 | LDL3 | LDL2 | LDL4 | mHDL | HDL1 | HDL2 |
| --- | --- | --- | --- | --- | --- | --- | --- | --- | --- |
| Protein (%) | 93 | 62 | 65 | 66 | 43 | 39 | 86 | 97 | 100 |
| Density | 1.43 | 1.28 | 1.30 | 1.30 | 1.20 | 1.17 | 1.40 | 1.46 | 1.47 |
| Conc. (nM) | 0.78 | 67 | 210 | 820 | 2,200 | 880 | 2,600 | 28,000 | 45,000 |

**S9 Table. Composition assuming that the anti-proteases do not form complexes with LDL.** LDL3 and LDL4 are particles with the same size as LAC1 and LAC2, respectively.
